# Extensive conformational and physical plasticity protects HER2-HER3 tumorigenic signaling

**DOI:** 10.1016/j.celrep.2021.110285

**Published:** 2022-02-01

**Authors:** Marcia R. Campbell, Ana Ruiz-Saenz, Yuntian Zhang, Elliott Peterson, Veronica Steri, Julie Oeffinger, Maryjo Sampang, Natalia Jura, Mark M. Moasser

**Affiliations:** 1 Department of Medicine, University of California, San Francisco, San Francisco, CA 94143, USA; 2 Departments of Cell Biology & Medical Oncology, Erasmus Medical Center, Rotterdam, the Netherlands; 3 Cardiovascular Research Institute, University of California, San Francisco, San Francisco, CA 94143, USA; 4 Department of Cellular and Molecular Pharmacology, University of California, San Francisco, San Francisco, CA 94143, USA; 5 Helen Diller Family Comprehensive Cancer Center, University of California, San Francisco, San Francisco, CA 94143, USA; 6 Lead contact

## Abstract

Surface-targeting biotherapeutic agents have been successful in treating HER2-amplified cancers through immunostimulation or chemodelivery but have failed to produce effective inhibitors of constitutive HER2-HER3 signaling. We report an extensive structure-function analysis of this tumor driver, revealing complete uncoupling of intracellular signaling and tumorigenic function from regulation or constraints from their extracellular domains (ECDs). The canonical HER3 ECD conformational changes and exposure of the dimerization interface are nonessential, and the entire ECDs of HER2 and HER3 are redundant for tumorigenic signaling. Restricting the proximation of partner ECDs with bulk and steric clash through extremely disruptive receptor engineering leaves tumorigenic signaling unperturbed. This is likely due to considerable conformational flexibilities across the span of these receptor molecules and substantial undulations in the plane of the plasma membrane, none of which had been foreseen as impediments to targeting strategies. The massive overexpression of HER2 functionally and physically uncouples intracellular signaling from extracellular constraints.

## In brief

Campbell et al. undertake a structure-function analysis of HER2-HER3 signaling when HER2 is overexpressed in cancers. They show that receptor and membrane topologies are much more flexible than had been envisioned and that the extracellular domains are not effective targets for disrupting intracellular HER2-HER3 signaling, which is driven entirely by overexpression.

## Graphical abstract

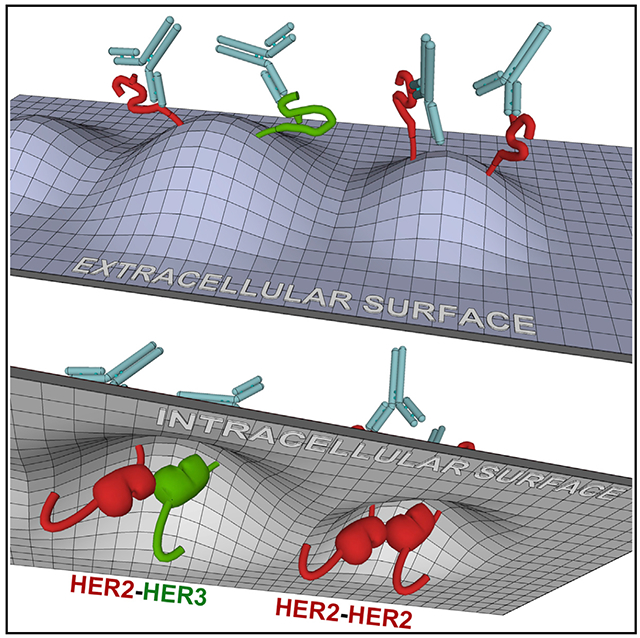

## INTRODUCTION

Aberrant activation of membrane-spanning receptor tyrosine kinases underlies the pathogenesis of many types of human cancers. The large extracellular domains (ECDs) of these receptors provide an ideal target site for biotherapeutic agents rationally designed for functional disruption through interfering with ligand-induced conformational changes or homotypic or heterotypic interactions. The highly selective and favorable safety and pharmacokinetic profiles of ECD-targeting agents and well-established discovery platforms and manufacturing processes have made ECD-targeting agents a mainstay of cancer therapy. However, this approach has been particularly challenging in the case of human epidermal growth factor receptor 2 (HER2)-amplified cancers. These tumors have massive overexpression of the oncogenic receptor HER2, resulting in its constitutive autophosphorylation, transphosphorylation of its essential partner HER3, and consequent activation of downstream signaling pathways that drive tumorigenic growth (Holbro et al., 2003; Moasser, 2007; Stern, 2008). The HER2-targeting antibody trastuzumab was developed in an era prior to structural information but is now known to bind to the juxtamembrane region of the HER2 ECD, where, in theory, it could interfere with proper engagement of partnering transmembrane domains (Cho et al., 2003). The HER2-targeting antibody pertuzumab, developed a decade later, was rationally designed to bind the dimerization interface of the HER2 ECD, interfering with HER2 homodimerization or HER2-HER3 heterodimerization (Agus et al., 2002; Franklin et al., 2004). Pertuzumab does indeed interfere with ligand-induced HER2-HER3 signaling, consistent with its structure-based mechanism of action (Figure S1A; Agus et al., 2002; Sakai et al., 2007), but neither trastuzumab or pertuzumab are effective at disrupting constitutive HER2 signaling in HER2-amplified cancer cells (Figure S1B). The abundant binding of these antibodies to the surface of cancer cells does elicit anti-tumor immunologic activities *in vivo*, which underlies their modest but beneficial clinical activities (Bellati et al., 2010; Bianchini and Gianni, 2014; Gianni, 2008; Park et al., 2010; Perez et al., 2015; Taylor et al., 2007). The abundant binding of these antibodies also allows delivery of chemotherapeutic attachments with good tumoricidal activity and significant clinical utility (Burris et al., 2011; Lewis Phillips et al., 2008). Investigational antibodies targeting the ECD of HER3 are similarly effective at inhibiting ligand-driven physiologic HER2-HER3 signaling but are ineffective at disrupting HER2-HER3 signaling or inhibiting the growth of HER2-overexpressing cancer cells (Blackburn et al., 2012; Garner et al., 2013; Kugel et al., 2014; McDonagh et al., 2012; Schoeberl et al., 2010). Disrupting constitutive HER2-HER3 signaling may require more precise targeting approaches based on a deeper understanding of signal generation within this tumor driver complex. We performed a structure-function analysis of HER2-HER3 signaling to better understand whether and how its constitutive signaling activity is coupled with physical or conformational states of its ECD regions. This was undertaken to better inform the design of future biotherapeutic agents targeting the ECD, but the evidence revealed uncoupling of the ECDs from intracellular signaling activity in this disease state.

## RESULTS

**Figure 1.**
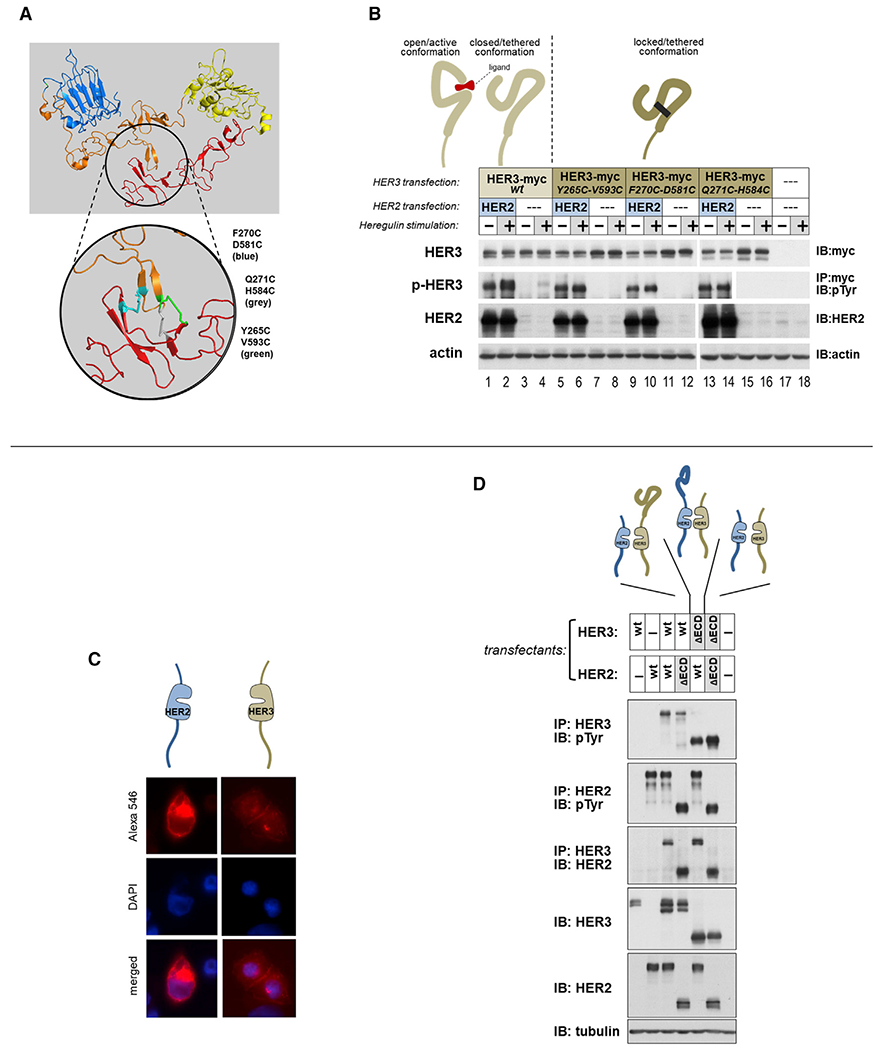
Constitutive HER2-HER3 signaling does not require canonical ECD-driven dimerization-inducing events (A) The ECD of HER3 was locked in the closed/tethered conformation by introducing double cysteine mutations at the indicated nearby residues of domains II and IV. Three different locked versions of HER3 were generated by mutating the indicated pairs of residues to cysteines as indicated. Of these, the Y265C/V593C double mutant has been studied extensively before and confirmed to be locked in the closed conformation by disulfide bridging (Kani et al., 2005). (B)CHO-K1 cells were transfected to express HER2 and HER3 mutants as indicated. Wild-type HER3 is constitutively phosphorylated in the presence of overexpressed HER2 (lane 1)and is further inducible by ligand stimulation (lane 2). There is also slight induction of HER3 phosphorylation by the background low level of endogenous HER2 in CHO-K1 cells (lane 4). The locked HER3 mutants, in the presence of overexpressed HER2, are fully competent at constitutive phosphorylation despite the fact that they are not able to adopt the open conformation and expose their dimerization interface and have lost ligand responsiveness (lanes 5–16). (C) The entire ECDs of HER2 and HER3 were deleted, preserving the N-terminal signal sequences. Membrane localization of these constructs was confirmed by immunofluorescence staining as indicated. (D) These constructs are fully competent at constitutive phosphorylation when HER2 is overexpressed, despite complete loss of ECD functions.

Massive HER2 expression most likely causes events, interactions, and consequences not encountered during the course of physiologic signaling at normal receptor levels. Therefore we reconstructed the pathological state of massive HER2 overexpression and constitutive HER2-HER3 signaling in an experimental Chinese hamster ovary (CHO)-K1 cell expression system with high transfection efficiency and at levels that mimic HER2-amplified cancer cells (Figures S2A and S2B). This system shows that, at a certain threshold level of HER2 overexpression, the ligand-independent constitutive phosphorylation of HER3 becomes evident. Normal physiological signaling is initiated by the interaction of HER3 with ligands, leading to conformational rearrangement of its ECD, exposing a dimerization interface that couples with a complementary interface on the ECD of HER2; the receptor proximation ultimately leads to dimerization of the intracellular kinase domains, and receptor phosphorylation ensues (Kovacs et al., 2015; Mitchell et al., 2018; Roskoski, 2014). Whether ligand stimulation has a driving role in HER2-amplified cancers has been debated but not conclusively determined. Even in the absence of ligand, by sheer abundance, the HER2 ECD may function to stabilize the open active conformation of the HER3 ECD in lieu of ligands. These concepts form the mechanistic bases for ECD-targeting antibodies designed to disrupt ligand binding, to lock the HER3 ECD in the closed/tethered conformation, or to bind and block the dimerization interfaces. To test these mechanistic hypotheses, we engineered three different HER3 constructs that are locked in the closed/tethered conformation of the ECD because of disulfide bridging (Figure 1A). These mutants lack ligand responsiveness but are fully competent at constitutive phosphorylation when HER2 is overexpressed, eliminating the role of ligand or non-ligand stabilization of the HER3 open/active conformation in the observed constitutive signaling (Figure 1B). Because there may yet be uncharacterized ECD interaction interfaces that mediate the signaling activity observed in the state of HER2 overexpression, we deleted the ECDs of HER3 or HER2 or both, but constitutive HER3 phosphorylation in the state of HER2 overexpression persisted despite the absence of ECDs (Figures 1C and 1D). It is evident that HER2-HER3 engagement and phosphorylation in the state of HER2 overexpression are not driven by the canonical ECD-driven dimerizing forces.

**Figure 2.**
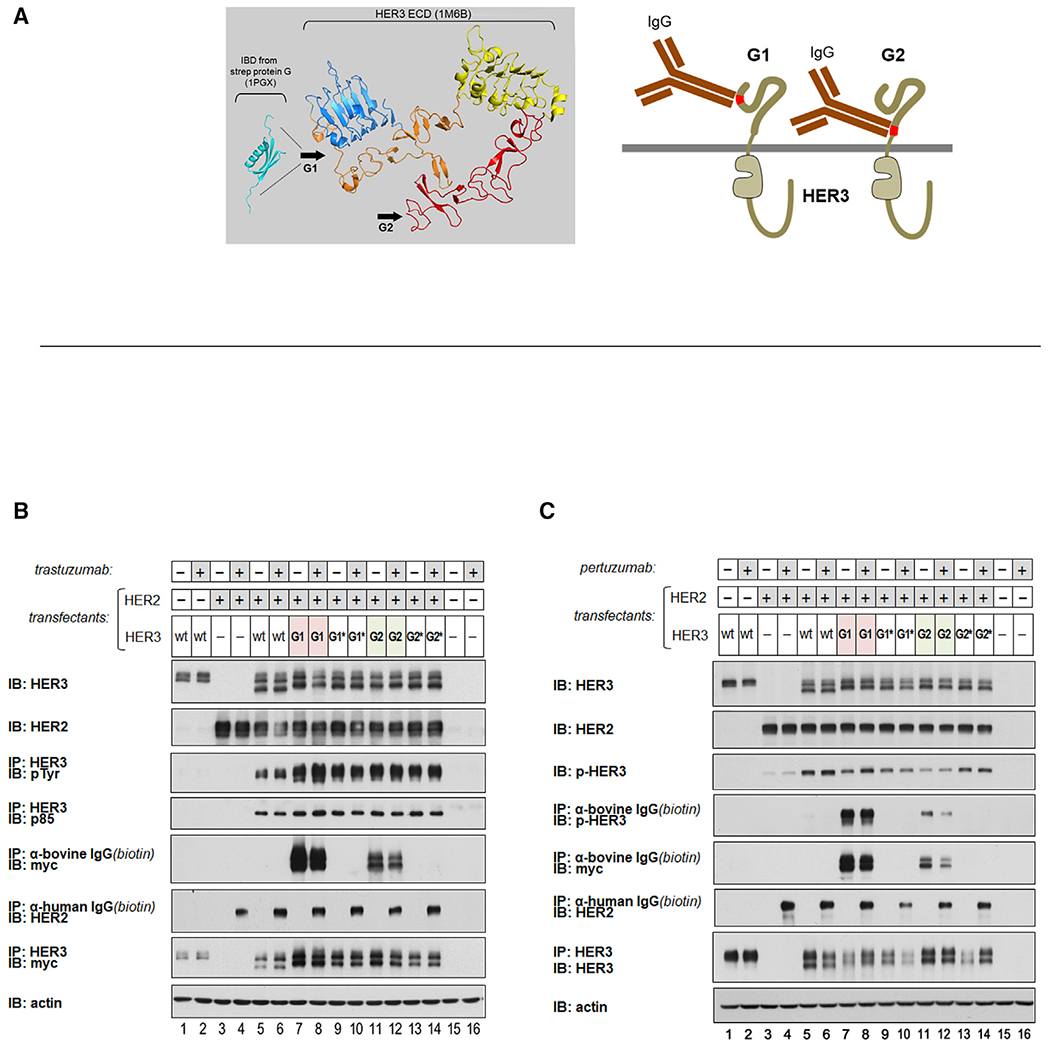
Constitutive HER2-HER3 signaling cannot be inhibited by proximity-restricting ECD-binding antibodies (A) High affinity IgG binding residues from the Ig-binding domain of streptococcal protein G were inserted into the indicated G1 or G2 linker regions of the HER3 ECD. (B) The HER3-G1 and HER3-G2 mutants were expressed in CHO-K1 cells along with overexpressed HER2 and assayed as shown. The altered HER3 constructs are myc tagged. When cultured in medium, the abundant IgGs in bovine serum bind the engineered HER3 constructs. The G1* and G2* constructs are negative control versions of the G1 and G2 constructs, mutated within the protein G sequence to abolish IgG binding. Despite confirmation of IgG binding, these proximity-restricting HER3 mutants are fully capable of constitutive HER2-HER3 phosphorylation in CHO-K1 cells when HER2 is overexpressed. The double banding of HER3 is due to glycosylation effects (Figure S2C). (C) The experiment was repeated using pertuzumab, which binds HER2 at its dimerization interface.

Although ECD interactions may not be driving constitutive signaling in HER2-amplified cancers, the ECDs may yet provide suitable targets for antibodies or other large biotherapeutic agents that add bulk to these receptors. By introducing steric clash and restricting receptor proximation, they could impede the kinase domain interactions that are essential for signal generation. Such proximity-restricting therapeutic agents would require careful positioning of the agent for maximal effect. We modeled and tested the potential in proximity-restricting interventions through receptor engineering. We introduced high-affinity immunoglobulin G (IgG)-binding residues into linker regions of the HER3 ECD, where it would not disrupt the structured domains. These epitopes were inserted at the domain I-II linker of HER3, where IgG binding could maximally clash with the extended ECD regions of HER2, or at the juxtamembrane region following domain IV of HER3, where it could maximally clash with proximation of the transmembrane domains (Figure 2A). However, such precise binding of IgG molecules to HER2 and HER3 does not abate the constitutive HER3 phosphorylation that occurs in the state of HER2 overexpression (Figures 2B and 2C).

**Figure 3.**
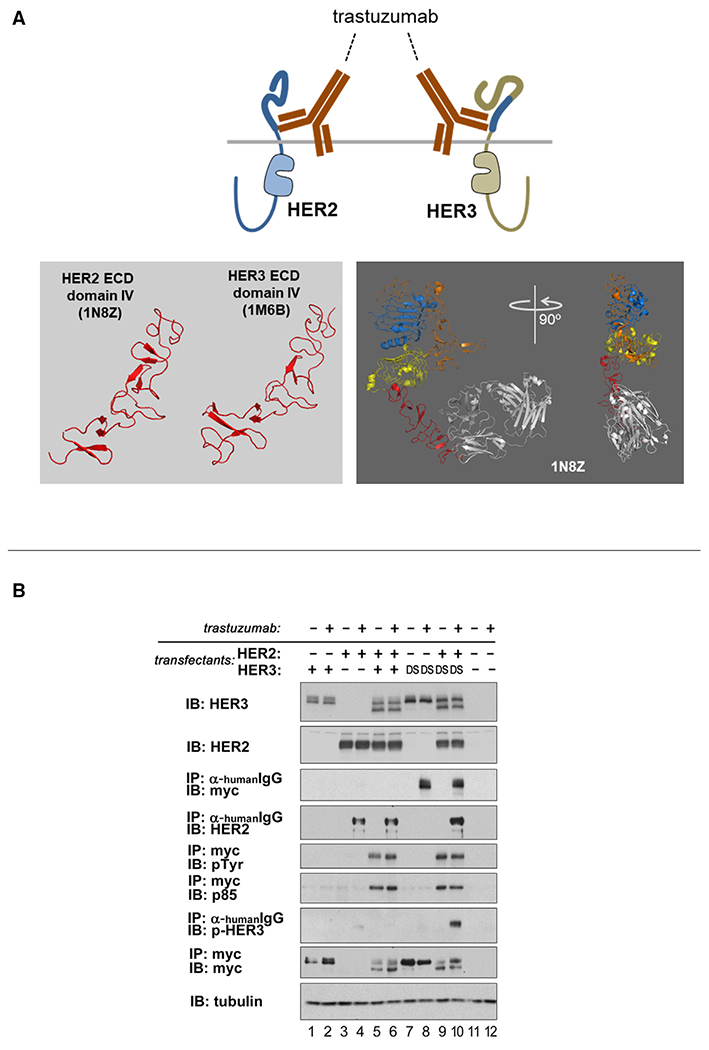
Constitutive HER2-HER3 signaling persists despite optimized in-plane interference with ECD proximation (A) Domain 4 of the HER3 ECD was replaced with domain 4 of the HER2 ECD, creating the domain-swapped (DS) mutant of HER3. These domains are structurally homologous, but the transposition brings the trastuzumab-binding epitope to the juxtamembrane region of HER3. Trastuzumab binds its target in a conformation where it protrudes directly into the dimerization plane (PDB: 1N8Z; Cho et al., 2003), creating considerable steric clash within the dimerization plane between its two targets that would be maximally restrictive to receptor proximation. (B) Despite confirmed trastuzumab binding to HER2 and the HER3-DS mutant, constitutive HER2-HER3 signaling persists in this engineered scenario in CHO-K1 cells.

Although inserting IgG target residues on the ECD can dictate the precise binding site, it cannot dictate the orientation of IgG binding. The orientation of binding would be an important determinant of steric clash but is not readily predictable. To engineer IgG targeting with intentionally designed orientation, we took advantage of known crystal structures of antibody-bound HER2. Pertuzumab binds HER2 at domain II but at an angle to the dimerization plane (Franklin et al., 2004). However, trastuzumab binds HER2 at domain IV in an orientation that directly protrudes into the dimerization plane, a better conformation for creating steric clash (Cho et al., 2003; Figure 3A). To take advantage of this known binding conformation, we replaced domain IV of the HER3 ECD with domain IV of the HER2 ECD. These regions of the HER2 and HER3 ECDs are structurally homologous and not expected to disrupt the overall architecture of the ECD (Figure 3A). This alteration brings the high-affinity trastuzumab binding epitope onto HER3 and creates a scenario where trastuzumab binds HER2 and HER3 near the transmembrane region, with both IgGs protruding directly into the dimerization plane, creating steric clash that would maximally restrict receptor proximation and kinase domain dimerization and signaling. However, the constitutive phosphorylation of HER3 persists despite confirmed binding of trastuzumab to HER2 and modified HER3 (Figure 3B).

**Figure 4.**
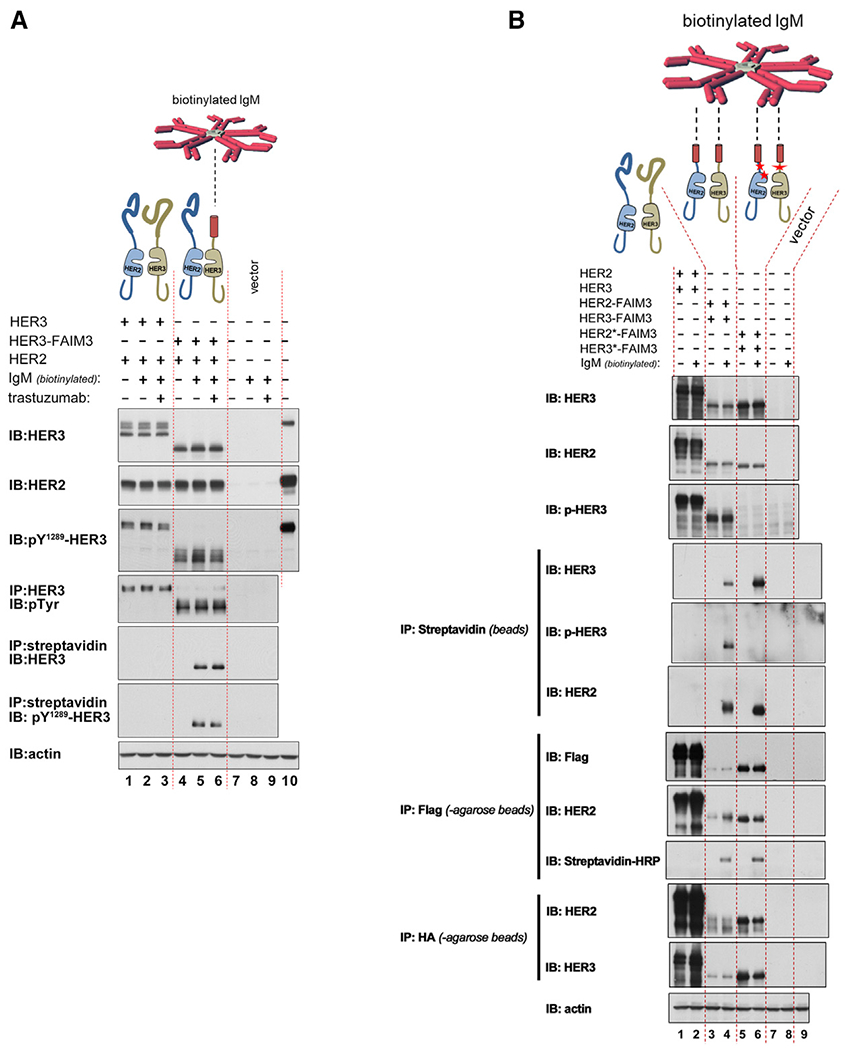
Constitutive HER2-HER3 signaling persists despite Elizabethan collar restrictions on ECD proximation (A) The ECD of HER3 was replaced with the ECD of FAIM3, the receptor for pentameric IgM, and expressed along with overexpressed HER2 in CHO-K1 cells. Despite treatment with biotinylated pentameric IgM and confirmed IgM binding to HER3, constitutive HER2-HER3 signaling persists. SkBr3 lysates were used in lane 10. (B) The ECDs of HER2 and HER3 were replaced with the ECD of FAIM3, and these hybrid receptors were expressed in CHO-K1 cells and treated with biotinylated pentameric IgM. Despite treatment with biotinylated pentameric IgM, constitutive signaling persists. The HER2* and HER3* versions in lanes 5 and 6 are negative control mutants that have additional mutations in the intracellular region that disrupt kinase domain dimerization and activation. These include mutations within the juxtamembrane regions of HER2 and HER3 and mutations in the N-lobe of the HER2 kinase domain. In lane 9, the lysates from lane 4 were used, but the pull-down control was with mouse IgG-agarose beads. The HER2 constructs are hemagglutinin (HA) tagged, and HER3 constructs are FLAG tagged.

These and other efforts to restrict receptor proximity and interaction were conceived based onto assumptions about the structures and conformations of these receptors. Although we typically conceptualize the HER family receptors as a planar assembly of individually characterized structures, the actual full-length structure may, in fact, not have such rigidity, and the ECDs may have rotational freedom with respect to the intracelluar domains (ICDs). This would mitigate efforts to disrupt signaling by single antibodies but leaves potential for use of cocktails of antibodies that can bind HER2 or HER3 in a multitude of orientations, affecting proximity restriction despite rotational freedom in the ECDs. We modeled this therapeutic approach by replacing the ECD of HER3 with the ECD of FAIM3, the receptor for pentameric IgM (Kubagawa et al., 2009; Shima et al., 2010). Binding of pentameric IgM to the extracellular regions of HER2 or HER3 provides a molecular microenvironment on the extracellular surface that is restrictive in all orientations, making it maximally intolerant to receptor proximation and transphosphorylation. However, despite confirmed binding of IgM to its extracellular region, HER3 is readily phosphorylated by overexpressed HER2 (Figures 4A and 4B). The persistent phosphorylation is not due to HER3 pools that are inaccessible to IgM because the precise biotinylated IgM-bound HER3 pulled down by streptavidin beads is found to be phosphorylated (Figure 4A, lanes 5 and 6). The surprising persistence of HER3 phosphorylation in these CHO-K1 cell structure-function studies is due to continued HER2-HER3 transphosphorylation and not due to a long phosphorylation half-life because, in the same assays, HER3 is dephosphorylated rapidly following lapatinib treatment (Figure S3).

**Figure 5.**
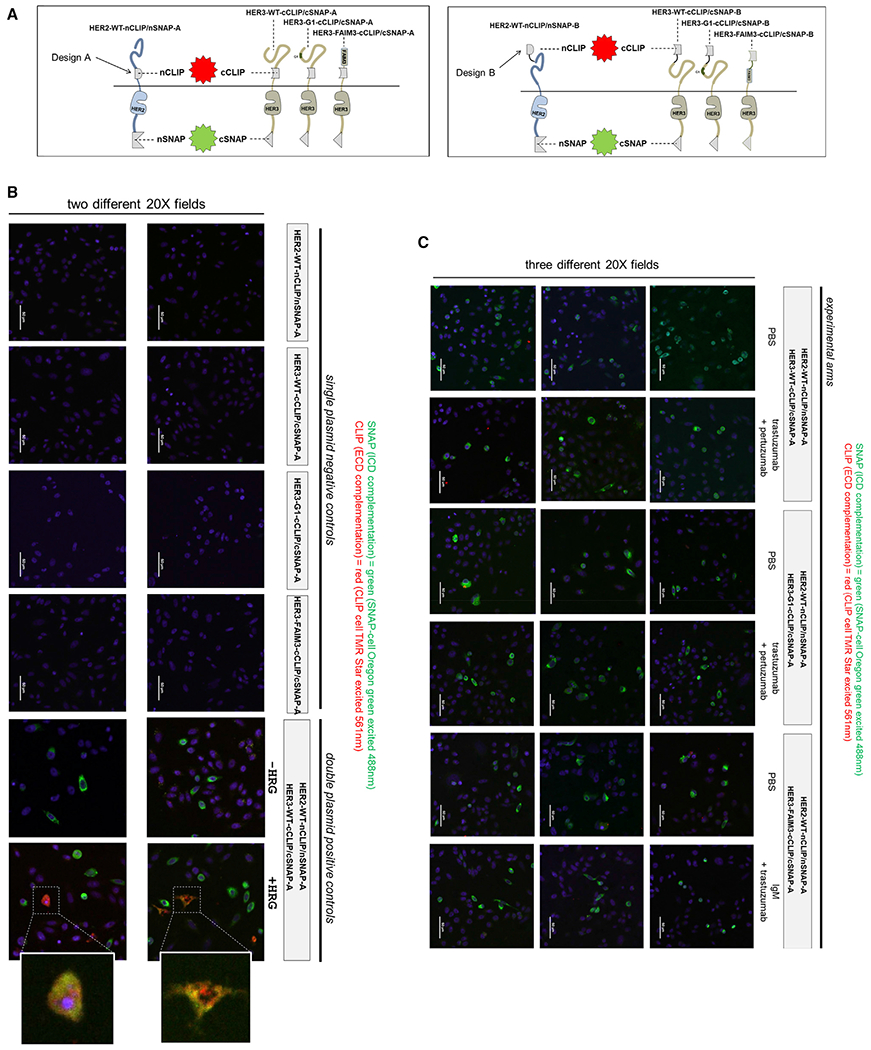
Resilient intracellular but not extracellular HER2-HER3 dimerization is readily evident in protein complementation assays (A) Schematic of the engineered constructs. Complementation of the split CLIP fragments is detected using CLIP substrates emitting red fluorescence, whereas complementation of the SNAP fragments is detected using SNAP substrates emitting green fluorescence. Because the CLIP fragments can potentially interfere with ECD dimerization, two different engineering designs (A and B) were employed, positioning this fragment at the N-terminus or at the juxtamembrane region of the ECD. CHO cells were transfected with the indicated constructs. Complementation of the intracellular SNAP tags was visualized using SNAP-cell Oregon green (green fluorescence), and complementation of the extracellular CLIP tags was visualized using CLIP-cell TMR-Star (red fluorescence) and using confocal microscopy with the appropriate laser excitation and filters. Blue fluorescence indicates Hoechst staining of cell nuclei. (B) Single-transfectant negative controls and baseline and ligand-stimulated cotransfection positive controls. (C) The various antibody-treated experimental arms and their untreated controls. Experiments with design (A) are shown here, and experiments with design B constructs are shown in Figure S5.

In an orthogonal approach to look at the dimerization of the ECDs and ICDs of these receptors, we performed functional assays of dimerization using protein fragment complementation techniques that generate fluorescent reporter signals. This is based on complementation of the split SNAP and CLIP tags as described previously (Mie et al., 2012, 2016). The HER2 and HER3 receptors were engineered to encode complementary split SNAP tags within the ICDs at the C-terminal end and complementary split CLIP tags within the ECDs. In two alternate construct designs, the split CLIP tag within the ECD was positioned at the juxtamembrane region following domain 4 of the ECD (design A) or at the N-terminal end of the receptors preceding domain I (design B) (Figure 5A). These reporter designs were engineered onto the wild-type HER2 and HER3 receptors as well as the HER3-G1 and HER3-FAIM3 constructs that were described previously. When transfected into cells, dimerization of the ECDs and the ICDs could be assayed simultaneously using SNAP and CLIP substrates with different fluorescence emission properties. Cotransfection assays were performed in CHO-K1 cells and visualized by confocal fluorescence microscopy. The intact full-length expression of these artificial constructs was confirmed by immunoblotting (Figure S4). These experiments show constitutive ICD dimerization in all co-transfection assays, but constitutive ECD dimerization is much less abundant, although it can be induced by ligand stimulation (Figures 5B and 5C). This is consistent with the fact that receptor overexpression readily drives kinase domain dimerization and signaling, as seen in the immunoblotting assays described previously. These assays go further by suggesting that, unlike kinase domain (KD) dimerization, ECD dimerization is not constitutively engaged in this overexpression-driven disease state. Although it is possible that the lack of observed constitutive ECD dimerization is due to artificial insertion of the CLIP fragments, which may disfavor dimerization, this is unlikely because similar results are obtained with different positioning of the CLIP tags (design B, Figure S5). In addition, these artificial constructs are, in fact, competent at ECD dimerization, as seen by positive control arms using ligand stimulation (Figures 5B and S5). Furthermore, the complementation of the extracellular CLIP tags does not necessarily require functional ECD competency and proper dimerization; rather, it merely requires ECD proximity, and such ECD proximity appears to be lacking in most instances of ICD dimerization. Because cellular green autofluorescence can create considerable background in some cells, these experiments have been repeated in both permutations using green SNAP and red CLIP substrates and also in a reverse assignment using far-red SNAP and green CLIP substrates (Figures 5 and S5).

Our finding that constitutive ICD dimerization, but not ECD dimerization, is readily detectable when HER2 is overexpressed is entirely consistent with other techniques used for detecting dimers. Proximity ligation assays (PLAs) frequently used to detect HER2-HER2 and HER2-HER3 dimers in breast cancers require probes targeting the intracellular domain (not ECD) of these receptors (Banappagari et al., 2013; Barros et al., 2014; Green et al., 2014; Kanthala et al., 2014; Spears et al., 2012). In addition, the whole-cell (including intracellular) localization of HER2-HER3 dimers seen in our SNAP complementation system replicates the observations from PLA and fluorescence resonance energy transfer (FRET)-based assays of HER2-HER3 dimers performed on formalin-fixed paraffin-embedded (FFPE) sections of HER2-amplified cancers (Barros et al., 2014; Fichter et al., 2014; Green et al., 2014; Kennedy et al., 2019; Weitsman et al., 2016). The whole-cell localization, including intracellular localization of HER2-HER3 dimers, reflects the well-known dual compartment localization and endosomal recycling of dimers that occurs with HER2 and HER3 (Lenferink et al., 1998; Pietila et al., 2019; Waterman et al., 1998; Worthylake et al., 1999). Even antibody-bound or designed ankyrin-repeat protein (DARPin)-bound HER2 receptors have been shown to internalize and recycle back to the surface with the biotherapeutic agents still attached (Austin et al., 2004; Shilova et al., 2015). Our SNAP and CLIP assays are done in living cells with continued incubation for up to 30 min following completion of fluorophore labeling to allow washout and background reduction; thus, these assays reflect a tracing of continued protein trafficking beyond the moment of labeling and prior to fixation. These tags may also interfere with the dynamics of endosomal recycling, further distorting the trafficking dynamics of these receptors. The dimer localization findings are entirely consistent with many other studies using a variety of techniques, cells, and tissues and consistent with the current understanding of receptor trafficking in the HER family.

**Figure 6.**
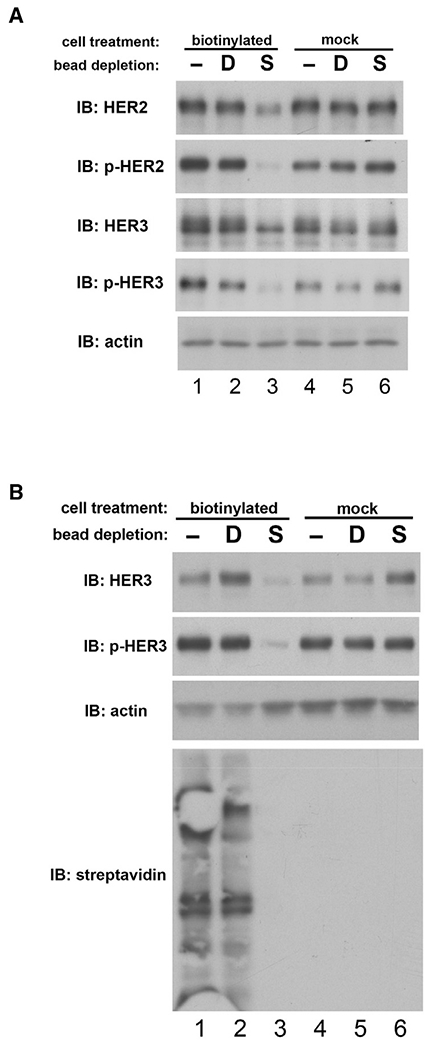
Constitutive HER3 signaling occurs almost entirely from the plasma membrane, not the intracellular pools (A) The surface of CHO cells expressing HER2 and HER3 was biotinylated using a cell-impermeable reagent. The entire surface proteome was then depleted from cell lysates using streptavidin beads, and HER2-HER3 expression and signaling activity was assayed in the intracellular lysate as shown. Lane 3 shows the membrane-depleted intracellular lysate; all other lanes are various negative controls. S indicates depletion by streptavidin beads, D indicates dummy beads, and – indicates no beads. (B) The same experiment was performed on HER2-amplified HCC1569 breast cancer cells. HER3 signaling in the intracellular lysate was assayed as shown. The streptavidin immunoblot shows the total depletion of the surface proteome in experimental lane 3.

HER2-HER3 dimers may or may not be phosphorylated and signaling throughout their endocytic recycling route and in theory, there may be parked intracellular pools that could generate signaling activity that is inaccessible to macromolecular biotherapeutic agents. We assayed the contribution of signaling from the intracellular compartment by stripping the membrane proteome of CHO-K1 cells expressing HER2 and HER3 using surface biotinylation techniques. Although there are intracellular pools of HER2 and HER3, there is very little phosphorylation within the intracellular compartment, and almost all of the signaling activity comes from the membrane compartment (Figure 6A). Similar findings are seen in HER2-amplified HCC1569 breast cancer cells (Figure 6B). Our observation that HER2-HER3 phosphorylation in HER2-amplified cancers is restricted to the membrane compartment is entirely consistent with observations from FFPE studies of HER2-amplified cancers (Bai et al., 2013; Frogne et al., 2009; Kanomata et al., 2019; Kurebayashi et al., 2015; Trono et al., 2016). The fact that receptor phosphorylation is seen predominantly at the plasma membrane does not conflict with the finding that physical dimers can be detected throughout the cell. Dimerization is the event that leads to phosphorylation, but the durability of these two biophysical and biochemical events follows different kinetics, and these two entities may begin simultaneously but do not parallel each other over time. Although dimers may be maintained during a cycle that traverses the endocytic trafficking within the intracellular compartment, phosphorylation is not maintained during receptor internalization. The reasons for this can include the predominantly intracellular localization of HER2- and HER3-targeting phosphatases, the action of chaperone complexes, or other factors (Gensler et al., 2004; Gijsen et al., 2012; Yuan et al., 2010; Zhu et al., 2008).

**Figure 7.**
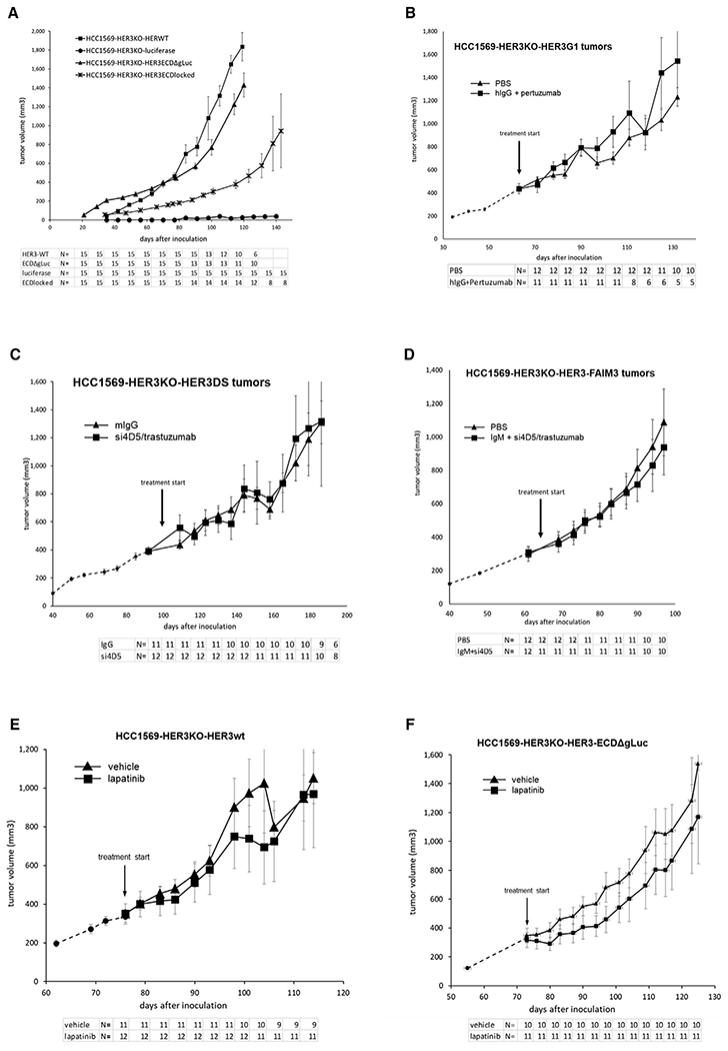
HER2-HER3-driven tumor growth in HER2-amplified cancer cells does not require ECD function and is not limited by proximity-restricting ECD-binding macromolecules (A) HER3 was knocked out in HCC1569 cells (HCC1569HER3KO) and replaced by the indicated add-back versions. These include wild-type HER3 (positive control), firefly luciferase (negative control), and a HER3 construct with the ECD locked in the inactive conformation (Y265C/V593C; ECDlocked), or with the ECD entirely deleted and replaced by *Gaussia* luciferase (ECDΔgLuc). These tumor cells were implanted subcutaneously, and tumor growth was monitored in NSG mice. Although the volumes of the HCC1569HER3KO-HER3-ECD-locked tumors appear lower than those of the HER3 wild type, this is due to altered biology. This tumor grows in a more flat and diffuse pattern with frequent ulceration and loss of mice. (B) HCC1569HER3KO cells were engineered to re-express a HER3 construct with high-affinity IgG-binding residues inserted between ECD domains I-II (HER3-G1) (Figure 2A). These tumors were grown subcutaneously in NSG mice and treated as indicated. IgG treatment targets HER3 at the engineered target site, and pertuzumab targets HER2 at its dimerization interface, but tumorigenic growth persists. (C) HCC1569HER3KO cells were engineered to re-express the DS (HER3DS) version of HER3 (Figure 3A), which brings the trastuzumab-binding epitope to the extracellular juxtamembrane region of HER3. Trastuzumab binds the extracellular juxtamembrane regions of HER2 and HER3 in these cells, but tumor growth is unaffected. The Fc-mutated version of trastuzumab (si4D5) is used here to minimize its immunologically mediated anti-tumor effects and focus on its function-disrupting effects. (D) HCC1569HER3KO cells were engineered to express the FAIM3-HER3 hybrid receptor (Figure 4A) and grown subcutaneously in NSG mice. Treatment with pentameric IgM and trastuzumab/si4D5 does not inhibit the growth of these tumors despite the significant spherical constraints on proximation of IgM-bound HER3 with HER2. (E and F) HCC1569 tumors with wild-type HER3 (E) or the ligand nonresponsive HER3 ECDΔgLuc hybrid mutant (F) were used here to inoculate the mammary fat pads of NSG mice. When tumors reached approximately 300 mm^3^, mice were randomized to receive treatment with lapatinib (80 mg/kg/day) in two divided doses by oral gavage or vehicle control. This dose of lapatinib is below the maximal tolerated dose of 100 mg/kg/day, which effectively suppresses HCC1569 tumor growth. A submaximal dose was used to best demonstrate the supersensitivity of the ligand-nonresponsive mutant if such an effect were to be seen. The ligand-nonresponsive mutant of HER3 shows no sensitization to lapatinib compared with the wild type. The numbers of surviving mice along the time course of the animal studies are shown for each arm underneath, and the sample size reduction over time in some arms reflects removal of mice for euthanasia because of large tumors, as mandated by guidelines. The error bars reflect SEM.

These cell-based expression systems in CHO-K1 cells provide an informative platform for structure-function studies with biochemical and biophysical readouts in an environment of massive HER2 overexpression mimicking HER2-amplified cancers. The relevance of these readouts to tumor growth was assayed more directly in HCC1569 HER2-amplified cancer cells by HER3 gene replacement techniques (Figure S6A). The failure of antibody therapeutic agents to disrupt intracellular signaling is also evident in *in vitro* studies of these engineered, stably transfected cells (Figure S6B). Although these HER2-amplified tumors cannot grow *in vivo* without functional HER3, they retain tumorigenic growth when the HER3 ECD is locked in the closed/tethered conformation or when the HER3 ECD is eliminated entirely (Figure 7A). Their growth is not inhibited by proximity-restricting therapeutic designs, including strategically targeted dual IgG binding (Figure 7B), dual juxtamembrane trastuzumab binding within the dimerization plane (Figure 7C), and Elizabethan collar-type restrictive IgM binding (Figure 7D).

*In vitro* experiments have suggested that constant ligand stimulation in the *in vivo* microenvironment may activate HER3 and promote tumor growth or mitigate the efficacy of HER2 inhibitors in HER2-amplified cancers (Claus et al., 2018; Leung et al., 2015; Sato et al., 2013; Wilson et al., 2012; Xia et al., 2013; Yang et al., 2017). This has never been studied directly *in vivo*. We used our HER3 gene replacement technique to test this hypothesis *in vivo*. Using a HER3 mutant lacking ECD ligand binding activity, we observe that tumor growth is not diminished (Figure 7A) and sensitivity to lapatinib is not increased (Figures 7E and 7F) by eliminating ligand binding, and it is evident that *in vitro* studies of ligand administration have overestimated the role of ligand stimulation *in vivo*.

## DISCUSSION

The fact that HER2-HER3 tumorigenic signaling persists despite restrictively re-engineering their ECDs challenges our assumptions about the conformational positioning and distribution of these receptors at the surface of cancer cells. The massive overexpression of HER2 appears to be sufficient to promote kinase domain interactions and generate constitutive signaling without the requirement for canonical dimerization-inducing functions of the ECDs and not limited by the conformational restraints linked with these bulky receptor regions. These observations reveal flexibility in the receptor structures themselves and suggest plasticity in the plane of the plasma membrane. We typically conceive and schematically draw these receptors as rigid structures (Figure S7A). These assumptions are derived from structural studies of purified fragments of the extracellular, transmembrane, and intracellular regions of these receptors using X-ray crystallography and NMR techniques. However, these structured modules are tied together by linking regions, and any assumptions regarding the conformation and rigidity of the whole receptor complex embedded in the plasma membrane remains speculative. The ECDs may indeed have rotational or angular flexibility with respect to the ICDs (Figure S7B; Video S1). Indeed, a comparison of the structures of monomeric and ligand-bound dimeric ECDs of epidermal growth factor receptor (EGFR) shows considerable flexibility and bending in the region linking the ECD with the transmembrane domain, with mutational studies confirming the absence of structural rigidity in this region (Lu et al., 2010). Also consistent with this, cryoelectron microscopy (cryo-EM) studies of purified EGFR reveal that the ECD and the kinase domain do not display a uniform orientation with respect to each other, consistent with the lack of conformational rigidity between the intracellular and extracellular regions of this receptor family (Mi et al., 2011). This rotational freedom in the ECDs can significantly mitigate the activity of biotherapeutic agents designed to restrict proximity. The uncoupling of ECD interactions from the kinase domains can be much more affected by the shape of the membrane. Although we conceptualize the cell membrane as a flat plane to envision receptor interactions, these conceptualizations are likely overly simplistic. Curvature in the cell membrane can allow overexpression-driven kinase domain dimerizations that leave their ECDs entirely distant and capable of binding antibodies or other large biotherapeutic agents without disruption of kinase domain interactions (Figures S7C and S7D; Video S2). The fact that the surface of cancer cells is highly irregular is well known from electron microscopy studies (Ishiwata et al., 2018; Nanou et al., 2018). There is more specific evidence of significant plasticity in the membrane contour of HER2-amplified cancer cells, including ruffles, folds, and protrusions such as filopodia, where HER2 is concentrated and across which intracellular kinase domains can interact without proximation of the ECDs (Chung et al., 2016; Hommelgaard et al., 2004). There are, in fact, direct studies of HER2 dimerization using quantum dot labeling and scanning electron microscopy in HER2-amplified cancer cells, revealing that HER2 dimers are concentrated in ruffled areas of the membrane and lacking in flat areas of the cell membrane (Peckys et al., 2015). Although the data presented here challenge the naive way in which we envision receptor structures and interactions, they merely reinforce what is now well established by many lines of data obtained through direct visualization approaches.

These studies identify functional and physical uncoupling of the ECDs from the ICDs, when driven by massive HER2 expression, that makes it difficult to inactivate signaling by targeting their ECDs. The most promising potential for ECD-targeting biotherapeutic agents for HER2-amplified cancers is through chemodelivery or immunostimulatory mechanisms afforded by the large surface expression of HER2 rather than through a signal inactivation mechanism. Strategies to more effectively inactivate HER2-HER3 signaling in these cancers must focus on the intracellular kinase domains. There is much more untapped potential in targeting the kinase domains of these receptors (Campbell et al., 2022 [this issue of *Cell Reports*]), which provide promising avenues for exploration. Although targeting ECDs may not be disruptive to KD dimerization and signaling, it may induce changes in how the KDs interact, and it remains possible that ECD targeting can enhance the effects of some KD-targeting approaches.

### Limitations of the study

This work reveals why ECD-targeting approaches have not been able to yield agents that can inactivate the constitutive HER2-HER3 signaling seen in HER2-amplified cancer cells. Although this suggests futility in this approach, it does leave room for some subtle effects that can be afforded by ECD-targeting agents. Although kinase domain dimerization and activation may persist in antibody-bound receptors, it is possible that antibody binding to the ECD induces some subtle changes in how kinase domains dimerize with each other and that these subtle changes may alter their affinities for small-molecule kinase domain inhibitors. Furthermore, although this work has focused on receptor phosphorylation, downstream signaling, and tumorigenic growth as endpoints, it remains possible that interactions of these receptors with certain proteins are, in fact, disrupted by ECD targeting but with biologic effects that are more subtle and not detected here. Future work can explore these subtle effects of ECD targeting more directly.

## STAR★METHODS

### RESOURCE AVAILABILITY

#### Lead contact

Further information and requests for reagents should be directed to and will be fulfilled by the lead contact, Mark Moasser (mark.moasser@ucsf.edu).

#### Materials availability

Plasmids generated in this study are available from the lead contact upon request. The modified cell lines are available from the lead contact upon request under a material transfer agreement.

#### Data and code availability

- Data reported in this paper will be shared by the lead contact upon request.
- This paper does not report original code.
- Any additional information required to reanalyze the data reported in this paper is available from the lead contact upon request.

### EXPERIMENTAL MODEL AND SUBJECT DETAILS

#### Animal studies

All animal experiments were approved by the Institutional Animal Care and Use Committee at UCSF (IACUC).

### METHOD DETAILS

#### Cell culture

All cells (HCC1569, SkBr3, and CHO-K1) were maintained at 37°C and 5% CO_2_. HCC1569 cells were grown in RPMI1640 media, SkBr3 in DMEM Hams:F12 media, and CHO-K1 cells in F12K media. All media was supplemented with 10% fetal bovine, penicillin, streptomycin, and L-glutamine. For use in *in vitro* experiments lapatinib was purified from tablets (Glaxosmithkline) as previously described (Amin et al., 2010). Ligand stimulation was done using 40 ng/ml heregulin β1 for 15 minutes. Clinical grade trastuzumab and pertuzumab were purchased from our hospital pharmacy (Genentech ™ Herceptin and Perjeta).

#### Protein lysate preparation and immunoblotting

Cells were lysed in modified RIPA (mRIPA) buffer (1% Na Deoxycholate, 0.1% SDS, 1% NP-40 detergent, 150 mM NaCl, 10 mM Na phosphate buffer) supplemented with leupeptin, aprotinin, phenylmethylsulfonyl fluoride, sodium vanadate and a phosphatase inhibitor cocktail, incubated on ice for 30 minutes and precleared at 14,000 rpm for 10 minutes at 4°C. Lysates were quantified using the Pierce BCA assay. 30-50ug of protein lysate was denatured by boiling for 8 minutes with laemmli sample buffer and separated on 7-10% polyacrylamide gels and transferred to PVDF membrane at 100 V for 1.5 hours in the cold room. Membranes were blocked in 3% bovine serum albumin (BSA) for 45 minutes and immunoblotted overnight at 4°C with the relevant primary antibodies and visualized using horseradish peroxidase conjugated secondary antibodies.

#### Transfection of CHO-K1 cells

To recapitulate the disease state of HER2 overexpression we used a CHO-K1 cell transient transfection model system. 10 cm petri dishes were seeded with 3.5 X10^6^ cells and transfected 24 hours later. A total of 7ug of DNA (4.5ug of HER2 and 2.5ug of HER3) and 21ul of Lipofectamine 2000 were combined according to manufacturer’s directions and added to cells that had been washed with PBS and primed with 3.5 ml optimem serum free media. After a 4 hour incubation at 37°C the transfection complexes were replaced with fresh F12K media (with/without 10% FBS depending on the experiment). The next day cells were either treated with drug and harvested or immediately lysed into 375-425ul of cold modified RIPA buffer. These experiments have been performed several times and in different variations to confirm reproducibility and generate publishing quality data.

#### Immunoprecipitation

For immunoprecipitation of exogenously expressed proteins from CHO-K1 cells, cells were washed 1 X with PBS, lysed in mRIPA lysis buffer (400ul/10 cm plate) so that the final concentration of proteins was between 2-5ug/ul. 150-600ug of protein lysate from each sample was brought up to a common volume, incubated with the targeting antibody. The mix was rotated overnight at 4°C and the following day immunoprecipitated using either Protein G Sepharose 4 fast flow beads or Streptavidin conjugated agarose beads at 4°C for 1-2 hours. The protein-antibody-bead complexes were pelleted at 8000 rpm for 2 minutes and washed with cold modified RIPA buffer three times. The precipitated complexes were resuspended in 35ul of 2 X laemmli buffer, boiled for 8-10 minutes and separated on polyacrylamide gels.

CHO-K1 cells transfected with the hybrid HER3-FAIM3 construct were treated with biotinylated IgM. This hybrid protein was immunoprecipitated using immobilized anti-Flag antibodies or when IgM-bound immunoprecipitated with streptavidin beads. Immunoblotting was done using previously described antibodies or streptavidin-HRP.

#### Plasmid cloning

Vector cloning was done using Gateway Cloning technology. HER2 and HER3 ORFs were cloned into entry vectors from Thermo-FisherScientific (pDONR221 or pENTR4) or pDONR223-HER3 (ERBB3) which was a gift from William Hahn & David Root (Addgene plasmid # 23874 ; http://n2t.net/addgene:23874 ; RRID: Addgene_23874). Destination vectors used were pcDNA-DEST40 (contains c-terminal V5-His tags), or in-house modified versions of this vector to express c-terminal 2XFlag tags (pDEST40-2XFlag) or 2XHA (pDEST40-2XHA) tags or nSNAP (pDEST40-nSNAP2XHA) or cSNAP (pDEST40-cSNAP2XFL) tags. Lentiviral infections were done using pLEX-ires-GFP, modified from pLEX_307 destination vector (gift from David Root; Addgene plasmid # 41392 ; http://n2t.net/addgene:41392 ; RRID:Addgene_41392) or pLenti-CMV/TO-Hygro DEST (gift from Eric Campeau & Paul Kaufman (Addgene plasmid # 17291 ; http://n2t.net/addgene:17291 ; RRID:Addgene_17291).

#### Generation of mutant constructs

Some mutant constructs were generated using mismatched primers and mutation-specific PCR conditions. Other constructs were generated using gene synthesis (Genewiz). To lock the HER3 ECD in the closed/tethered conformation, opposing residues at appropriate distance were mutated to cysteines to mediate disulfide bridging. At least one of these has previously been characterized in some depth and confirmed by protease protection assays to be locked in the closed conformation (Kani et al., 2005). In HER2 or HER3 constructs lacking the ECDs, the signal sequences were preserved in order to ensure proper localization. For the HER3 mutants engineered to bind IgG, 50AA sequence of the protein G immunoglobulin binding domain (IBD) was inserted into appropriately selected sites within linker regions between domains I and II or at the end of domain IV of the ECD, based on the crystal structure of HER3 (PDB 1M6B), such as to minimally interfere with ECD folding and configuration. Although not intended to disrupt HER3 function in the absence of bound IgG, the G2 mutant does have some deficiency in signaling and is less accessible to IgG binding than the G1 mutant. To generate the G1* and G2* negative control versions that are deficient at IgG-binding, we introduced mutations that disrupt IBD-IgG binding based on existing knowledge of the structure of IBD-IgG binding (Derrick and Wigley, 1994; Gallagher et al., 1994; Sloan and Hellinga, 1999). The domain swap mutant of HER3 was similarly designed to swap approximately 85% of domain IV of HER3 with that of HER2, choosing endpoints that would minimally interfere with secondary structure. The sequences of all the mutated and engineered constructs are provided in the Supplemental information sequence file. Amino acid numbering annotation used in this paper reflects the entire open reading frame including the signal sequence.

#### Generation of split CLIP/SNAP constructs for complementation assays

These protein complementation assays were designed following the previously described complementation of split SNAP and CLIP fragments (Mie et al., 2012, 2016). The complementary fragments consist of AA 1-91 (nSNAP and nCLIP) and AA 92-182 (cSNAP and cCLIP) of the full length SNAP and CLIP proteins. The CLIP fragments were engineered onto the ECD of HER2 (nCLIP) or HER3 (cCLIP) using gene synthesis. GS linkers (4-12 AAs) were used to flank the CLIP fragments from ECD sequences such as to provide flexibility and access to the complementary fragment. The HER2 and HER3 signal sequences were always preserved. The SNAP fragments were engineered onto the expression vector backbone such that they would express a C-terminal nSNAP or cSNAP fragment in-frame with the Gateway cloned insert and the HA or FLAG tags. The sequences of all these engineered constructs are provided in the Supplemental information sequence file.

#### Immunostaining assays

CHO-K1 cells were seeded onto glass cover slips in 12 well plates that had been coated with 1ug/ml of fibronectin and dried. The next day cells were transfected with tagged HER2-V5 or HER3-MycHis and allowed to recover overnight. In preparation of immunostaining, cells were rinsed with cold PBS, fixed in 4% paraformaldehyde for 10 minutes, permeabilized with 0.1% triton X-100 for 10 minutes and blocked in 2% BSA for 45 min. To detect HER2-V5 localization, cells were stained at room temperature for 1hr with a 1:500 dilution of V5 antibodies in 2% BSA and detected with anti-mouse Alexa 546 secondary. To detect HER3-Myc-His, cells were stained for 1hr with a 1:200 dilution of anti-cMyc antibody in 2% BSA and detected with anti-mouse Alexa 546 secondary. Slides were mounted in 10ul of Vectashield Antifade mounting medium with DAPI and examined by fluorescence microscopy.

#### Immunofluorescent SNAP/CLIP complementation assays

12-mm coverslips were placed into 12-well (3.5 cm2) plates and coated with a 0.10% gelatin/0.0025% poly-L-lysine solution for 15 minutes. The solution was aspirated, and the coverslips were air-dried for 1 hour. The coverslips were seeded with 10^5^ CHO-K1 cells and incubated for 18-24 hours in antibiotic-free media. The cells were washed with PBS, primed with Opti-MEM serum-free media, and then transfected with a total of 3.0 μg of DNA (1.0 μg of HER2 and 2.0 μg of HER3) and 2-5 μL Lipofectamine 2000 transfection reagent according to the manufacturer’s protocol. After 4 hours of incubation at 37°C, the transfection complexes were replaced with fresh F-12K media (with 10% FBS) and incubated for 18-24 hours at 37°C. For heregulin stimulation arms, the cells were serum starved for 4 hours and stimulated with 40 ng/ml heregulin β. For antibody treatment arms they were treated with trastuzumab (50 μg/mL), pertuzumab (50 μg/mL), or human IgM (30 μg/mL) for 4 hours while incubating at 37°C. The cells were then washed with PBS and incubated with the appropriate SNAP-tag and CLIP-tag labelling substrates. The substrates used in this study include SNAP-Cell Oregon Green and CLIP-Cell TMR-Star, or SNAP-Cell 647-SiR and CLIP-Cell 505. All substrates were diluted 1:200 from their concentrated stock solutions (in DMSO) in complete F-12K media, and placed on the cells for 1 hour at 37°C. Excess substrate was removed by gently washing the cells 3x in complete F-12K media and incubating in substrate-free media for an additional 30 minutes after washing (per the manufacturer’s recommendation). Following the SNAP- and CLIP-tag labeling substrate incubation, the CHO-K1 cells were fixed for 10 minutes with 4.0% paraformaldehyde at room temperature. The cells were washed with PBS, incubated in 5.0 μM Hoechst 33342 (as a nuclear counterstain) for 2 minutes, and washed again in PBS. The coverslips were gently dried and mounted onto glass microscope slides using ProLong Gold antifade reagent (without DAPI). The slides were protected from light and allowed to cure for 24 hours prior to imaging. Slides were prepared for long-term storage by sealing the coverslips with clear nail polish and storing at −20°C.

#### Confocal microscopy

Three instruments were used in this study with the indicated lenses and lasers. 1) Zeiss Spinning Disk Cell Observer inverted confocal microscope. Equipment used were a Plan-Apochromat 20x/0.80 Ph 2 M27 objective and a Plan-Apochromat 63x/1.40 oil objective and three laser lines (405 nm, 488 nm, 561 nm), and an X-Cite 120Q mercury lamp. The image acquisition, adjustment, and exportation were performed using the ZEN 3.1 lite software from Carl Zeiss Microscopy GmbH. 2) Nikon Ti inverted CSU-22 spinning disc confocal microscope. Equipment used were a Plan-Apochromat 20x/0.75 DIC M N2 objective and a Plan-Apochromat VC 60x/1.40 oil DIC N2 objective and three laser lines (405 nm, 488 nm, 561 nm). The image acquisition, adjustment, and exportation were performed using NIS Elements software. 3) Zeiss Confocal Laser-Scanning upright Microscope 780-LSM. Equipment used were a W Plan-Apochromat 20x/1.0 DIC (UV) VIS-IR M27 75 mm objective and a Plan-Apochromat 63x/1.40 Oil DIC M27 objective and three laser lines (405 nm, 488 nm, 633 nm). The image acquisition, adjustment, and exportation were performed using the ZEN 3.1 lite software from Carl Zeiss Microscopy GmbH. The fluorescent proteins used in this study were excited at the following wavelengths; SNAP-Cell Oregon Green (488 nm), CLIP-Cell 505 (488 nm), CLIP-Cell TMR-Star (561 nm), SNAP-Cell 647-SiR (633 nm). Hoechst dye (405 nm).

#### HCC1569 HER3 gene switch cell line generation

The deletion of HER3 in HCC1569 breast cancer cells was previously described (Ruiz-Saenz et al., 2018). Briefly, using CRISPR-Cas9 technology, we engineered the elimination of HER3 expression in HER2-amplified HCC1569 breast cancer cells (ATCC CRL-2330). Three independent HER3 knockout (HCC1569-HER3KO) clones were confirmed to lack HER3 protein expression and were selected for further analysis. These HCC1569KO cells maintain proliferative growth in monolayer cell culture, but are substantially deficient in tumorigenesis in mouse xenograft hosts. To eliminate the role of clonal growth characteristics in the replacement experiments, the three separate clones of HCC1569-HER3KO cells were mixed together to generate a polyclonal HCC1569-HER3KO cell line and this cell line was used as the parental cell line for the various add-back studies described in this paper.

To generate the various HER3 add-back cell lines, HCC1569-HER3KO cells were transduced with wild-type or mutant versions of HER3. Mutant versions of HER3 were cloned into Gateway entry vectors and shuttled into the pLEX-ires-GFP destination vector using lentivirus particles produced, concentrated and titered at the UCSF lentiviral core (https://viracore.ucsf.edu/). Briefly, viruses were produced in 10 cm petri dishes using jetPRIME transfection reagent and 3^rd^ generation packaging plasmids. 72hrs post transfection, viral supernatants were collected, filtered, concentrated, titered and frozen immediately at −80°C. Due to the very large size of the HER3 pLEX lentiviral plasmids (13.9 kB), care was taken when handling viral supernatants. For viral transduction, HCC1569HER3KO cells were seeded into one well of a 24-well plate. The next day cells were refreshed with fresh media for 6 hours before transduction. Virus was thawed at room temperature and brought up to 700ul with RPMI media. Virus with 1 X Transdux reagent was added to cells and incubated overnight at 32°C. The following day 300ul of fresh media was added and cells were transferred to 37°C for 6 hours. At the end of the day the virus containing mix was replaced with fresh RPMI media. 96-120hrs post transduction, cells were selected with 1ug/ml of puromycin for at least 2 weeks. Our pLEX lentiviral construct contains the puromycin resistance gene as well as an IRES eGFP viral backbone. Fluorescence activated cell sorting was used to isolate and pool eGFP positive cells and exogenous HER3 expression was confirmed by western blotting of the Myc-6XHis tag. Pooled cells were grown in 0.25ug/ml puromycin to maintain exogenous HER3 expression.

#### Mouse tumor growth assays

A total of 5X10^6^ HCC1569 cells in 100ul (50% matrigel:50% serum free media) were implanted subcutaneously or into the mammary fat pad (where indicated) into NOD scid gamma (NSG) mice. Tumor growth was measured weekly starting about 4 weeks post cell implantation or when tumors became large enough to measure. When tumors reached the maximum size allowed under our IACUC guidelines, mice were euthanized.

For mouse treatment pertuzumab was purchased from our hospital pharmacy (Genentech ™ Herceptin and Perjeta), and administered by intraperitoneal injection at 15/mg weekly. Mouse silent anti-HER2 clone 4D5 (named si4D5) is the Fc-mutated murine version of trastuzumab deficient at immunologic activity, and was custom ordered from Absolute Antibody and administered by intraperitoneal injection at 15/mg weekly. Mouse IgG, human IgG, and human IgM were administered by intraperitoneal injection at 15/mg once (IgG) or twice (IgM) twice per week.

### QUANTIFICATION AND STATISTICAL ANALYSIS

The quantitative mouse tumor volume data is shown as the mean value with error bars showing the standard error of the mean (SEM). The sample sizes are shown below each graph including the beginning sample size and the surviving sample size over the timecourse of the experiments. The graphs were plotted using Excel.

## Supplementary Material

1

2

3

## Figures and Tables

**Table:** KEY RESOURCES TABLE

REAGENT or RESOURCE	SOURCE	IDENTIFIER
Antibodies		
Trastuzumab, anti-HER2	Genentech	Herceptin
Pertuzumab, anti-HER2	Genentech	Perjeta
si4D5 anti-HER2 mAb	Absolute Antibody	ab01093-1.32
Mouse IgG	ThermoFisher	02-6502
Human IgG	ThermoFisher	31154
Human IgM	ThermoFisher	31146
Human IgM-biotin-SP	Jackson ImmunoResearch	009-060-012
anti-HER2	SantaCruz Biotechnology	C-18 #284
anti-HER3	SantaCruz Biotechnology	5A12 #81455
anti-HER3 biotin	SantaCruz Biotechnology	C-17/custom
anti-bovine IgG biotin	SantaCruz Biotechnology	2420
anti-myc	SantaCruz Biotechnology	9E10 #40
anti-actin	SantaCruz Biotechnology	I-19 #1616
anti-HA	SantaCruz Biotechnology	Y11 sc-805
anti-HA	SantaCruz Biotechnology	F-7 sc-7392
anti-p-HER3 Y1289	Cell Signaling Technology	4791
anti-p-HER2 Y1248	Cell Signaling Technology	2247
anti-myc	Cell Signaling Technology	71D0 #2278
anti-streptavidin	Cell Signaling Technology	3999
anti-Flag	Clontech	635691
anti-Flag beads	Clontech	635686
anti-V5	Invitrogen	R960-25
anti-rabbit IgG-HRP	GE Healthcare	NA9340
anti-mouse IgG-HRP	Cell Signaling Technology	7076
anti-goat IgG-HRP	SantaCruz Biotechnology	2020
anti-mouse IgG Alexa 546	Invitrogen	A11630
Chemicals, peptides, and recombinant proteins		
Lapatinib	GlaxoSmithKline	Tykerb
Fetal bovine serum	Gemini Bioproducts	100-106
Penicillin-Streptomycin-Glutamine	ThermoFisher	10378016
Heregulin β1	SigmaAldrich	H0786
Phosphatase inhibitor cocktail	Roche	04906845001
Lipofectamine 2000	ThermoFisher	11668500
JetPrime transfection reagent	Polyplus	101000015
Transdux reagent	SystemsBio	LV850A-1
Optimem media	ThermoFisher	31985070
protein G Sepharose 4 fast flow	GE Healthcare	17-0618-02
streptavidin conjugated agarose	SigmaAldrich	85881
streptavidin HRP	Cell Signaling Technology	3999
ProLong Gold antifade reagent	Life Technologies	P36930
Hoechst 33342	ThermoFisher	62249
VectaShield anti-fade with DAPI	Vector Laboratories	H-1200
SNAP Cell Oregon Green	New England Biolabs	S9104S
SNAP Cell 647-SiR	New England Biolabs	S9102S
CLIP Cell TMR Star	New England Biolabs	S9219S
CLIP Cell 505	New England Biolabs	S9217S
Paraformaldehyde	Alfa Aesar	43368
transdux reagent	SystemsBio	LV850A-1
Puromycin	ThermoFisher	A1113802
Critical commercial assays		
Pierce BCA assay	ThermoFisher	23227
QuikChange II Site-directed mutagenesis kit	Stratagene	200521
Experimental models: Cell lines		
Hamster: CHO-K1 Chinese hamster ovary cells	ATCC	CCL-61
Human: MCF-7 breast cancer cells	ATCC	HTB-22
Human: SkBr3 breast cancer cells	ATCC	HTB-30
Human: HCC1569 breast cancer cells	ATCC	CRL-2330
Experimental models: Organisms/strains		
NSG (NOD-scid IL2Rgamma^null^) mice	Jackson Labs	005557
Recombinant DNA		
pDONR221	ThermoFisher	12536017
pENTR4	ThermoFisher	A10465
pDONR223-HER3	gift from William Hahn & David Root - Addgene	23874
pDEST40-2XFL	ThermoFisher / modified	12274015
pDEST40-2XHA	ThermoFisher / modified	12274015
pDEST40-nSNAP2XHA	ThermoFisher / modified	12274015
pDEST40-cSNAP2XFL	ThermoFisher / modified	12274015
pLEX-IRES-GFP destination	modified from pLEX307, gift of David Root, Addgene	41392
pLenti-CMV/TO-Hygro-destination	gift of Eric Campeau & Paul Kaufman, Addgene	17291
Other		
glass slides	ThermoFisher	12-550-15

## Highlights

- Massive HER2 overexpression in cancers promotes HER2-HER3 kinase domain activation
- Extracellular domains and their ligands are redundant in HER2-overexpressing cancers
- Disrupting extracellular domain interactions does not block kinase transactivation
- The receptor and membrane topologies are more flexible than previously envisioned
