# Extensive conformational and physical plasticity protects HER2-HER3 tumorigenic signaling

**DOI:** 10.1016/j.celrep.2022.111338

**Published:** 2022-09-06

**Authors:** Marcia R. Campbell, Ana Ruiz-Saenz, Yuntian Zhang, Elliott Peterson, Veronica Steri, Julie Oeffinger, Maryjo Sampang, Natalia Jura, Mark M. Moasser

In the originally published version of this article, Methods S1 was missing from the supplemental information file due to a processing error. The complete supplemental information file now appears online with the article.

