# Extensive conformational and physical plasticity protects HER2-HER3 tumorigenic signaling

**DOI:** 10.1016/j.celrep.2026.117358

**Published:** 2026-05-12

**Authors:** Marcia R. Campbell, Ana Ruiz-Saenz, Yuntian Zhang, Elliott Peterson, Veronica Steri, Julie Oeffinger, Maryjo Sampang, Natalia Jura, Mark M. Moasser

A previous version of this correction mistakenly linked to the article “Targetable HER3 functions driving tumorigenic signaling in HER2-amplified cancers” (https://doi.org/10.1016/j.celrep.2021.110291). As indicated by this correction’s title, it refers to the article “Extensive conformational and physical plasticity protects HER2-HER3 tumorigenic signaling” (https://doi.org/10.1016/j.celrep.2021.110285), a related article by the same author group, and should have linked to that article rather than to its companion article. This has now been corrected online. *Cell Reports* apologizes for the error and the confusion created.

