# Supplementary material for: Extensive conformational and physical plasticity protects HER2-HER3 tumorigenic signaling
Source: Cell Rep. Author manuscript; Available in PMC 2022 Feb 23. (PMC8865943; doi:10.1016/j.celrep.2021.110285)
Supplement: 1 [file NIHMS1776709-supplement-1.pdf]

Cell Reports, Volume *38*

Supplemental information

Extensive conformational and physical plasticity protects HER2-HER3 tumorigenic signaling

Marcia R. Campbell, Ana Ruiz-Saenz, Yuntian Zhang, Elliott Peterson, Veronica Steri, Julie Oeffinger, Maryjo Sampang, Natalia Jura, and Mark M. Moasser

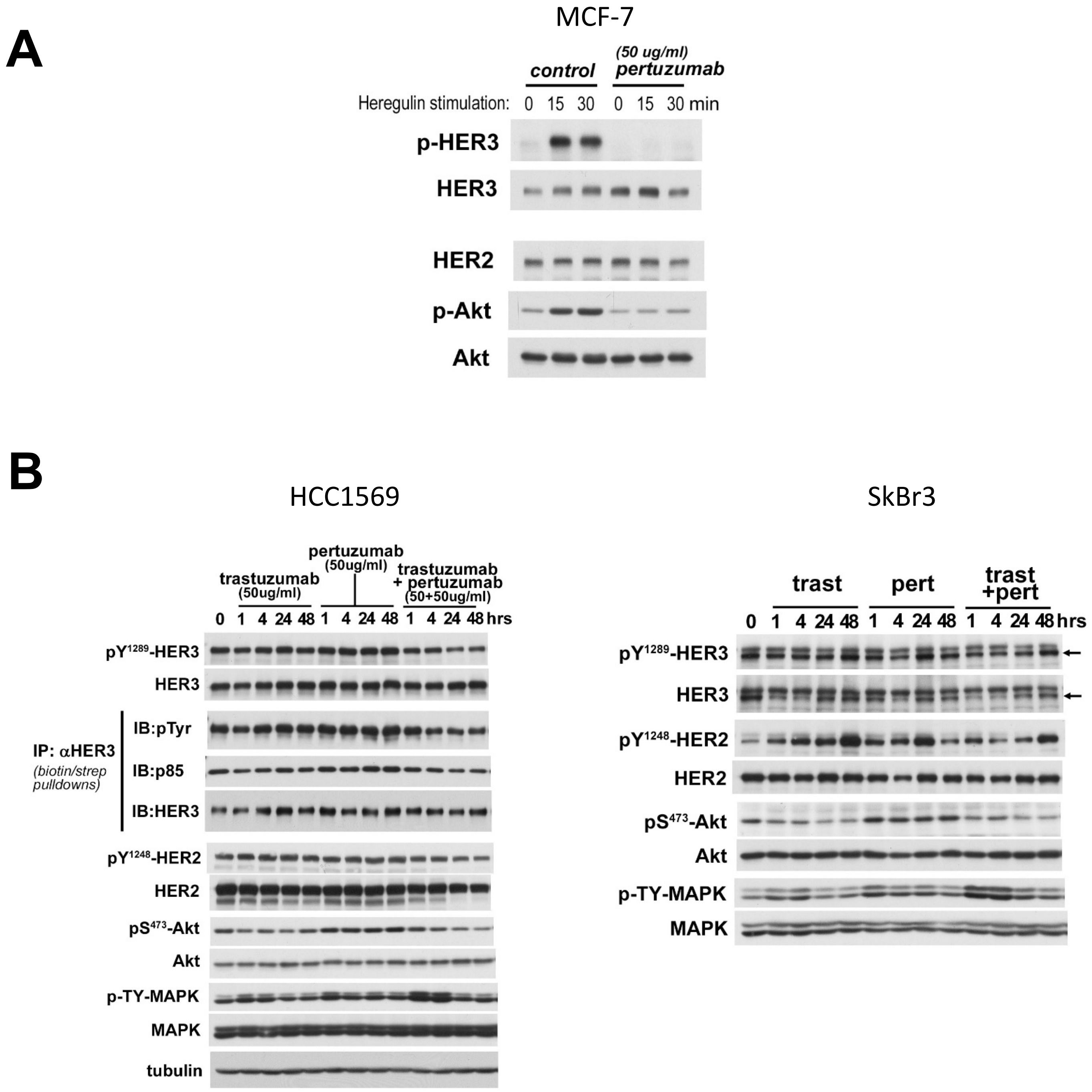

Figure S1. Trastuzumab and pertuzumab do not inactivate constitutive HER2 signaling in HER2-amplified cancer cells. Related to Figures 1,2,3,4.

A) MCF-7 breast cancer cells that have normal levels of HER2 were stimulated with 10ng/ml heregulin for 15-30 minutes with or without pertuzumab pretreatment and cell signaling assayed by western blotting as shown. **B)** HCC1569 or SkBr3 HER2-amplified breast cancer cells were treated with 50ug/ml of trastuzumab and/or pertuzumab for the indicated durations and cell signaling assayed by western blotting as shown.

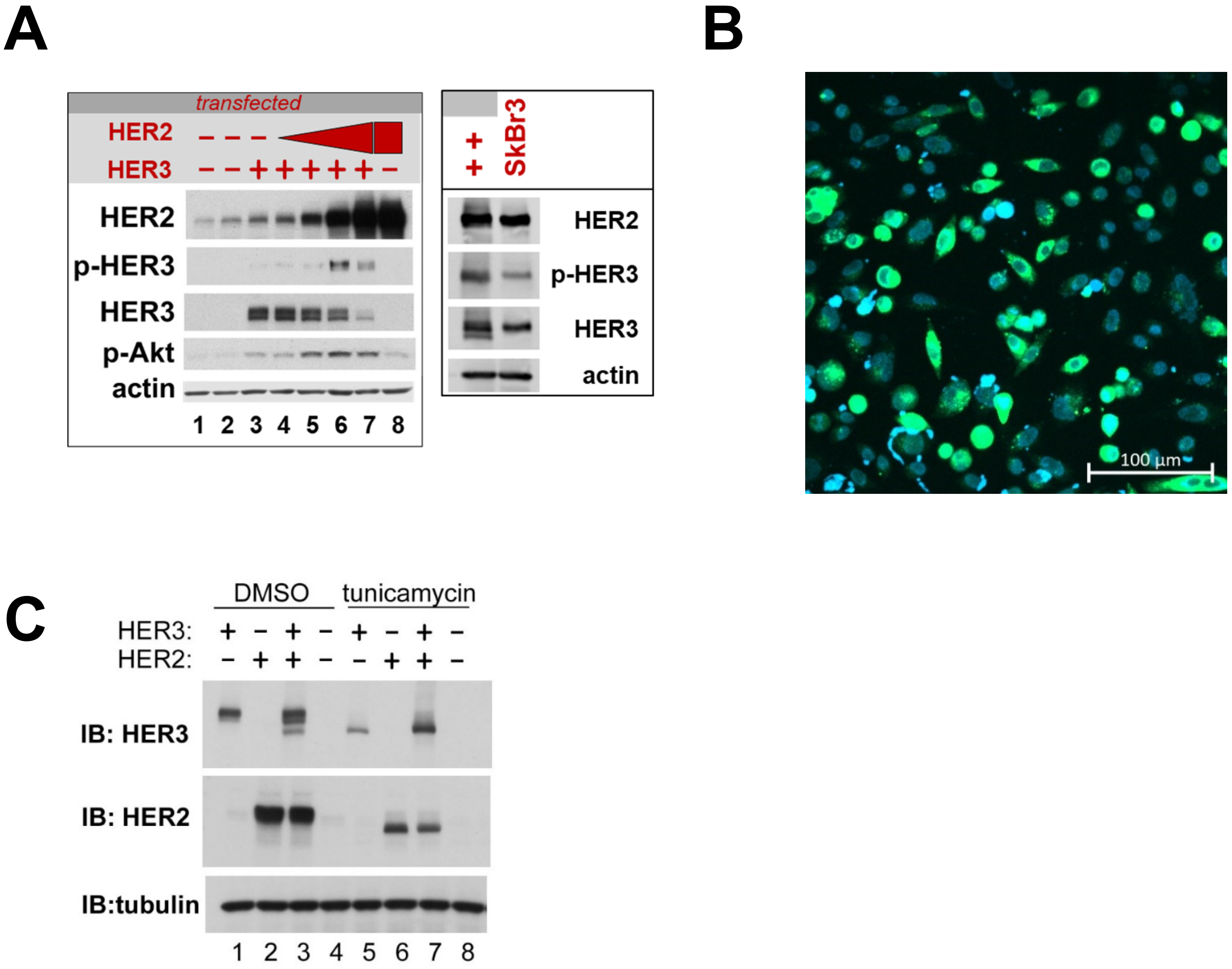

Figure S2. Experimental induction of HER2 overexpression in CHO cells. Related to Figures 1,2,3,4.
A) CHO cells were transfected with increasing amounts of pDEST40-HER2 and a fixed amount of pDEST40-HER3 and HER3 phosphorylation was assayed by western blotting following 12 hours of serum starvation as shown. The analysis shows constitutive HER3 phosphorylation at high levels of HER2 expression. The vector concentrations and expression levels of lane 6 were selected for the structure-function studies. The blot on the right shows the expression level of these transfected CHO cells side-by-side with lysates from HER2-amplified breast cancer cells.
B) CHO cells were transfected with pDEST40-HER2 with a c-terminal CLIP tag and the transfection efficiency was evaluated 24hrs later using CLIP-Cell 505 reagent and DAPI nuclear counterstain.
C) CHO cells were transfected with pDEST40-HER2 and a pDEST40-HER3 at previously established expression levels. Cells were treated with 1ug tunicamycin or control overnight and the cell lysates immunoblotted to observe HER2 or HER3 expression and migration sizes. When high levels of HER2 are co-expressed, HER3 migrates as a doublet in these cells (lane 3). This is due to glycosylation differences that become much more disparate only when HER2 is overexpressed. This is confirmed here by tunicamycin treatment which inhibits glycosylation and the associated double banding of HER3 (lane 7).

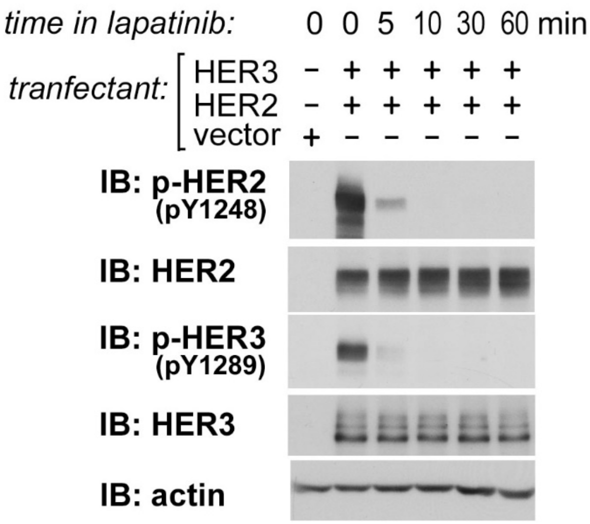

Figure S3. The phosphorylation half-lives of HER2 and HER3 are short. Related to Figures 1,2,3,4. CHO cells expressing HER3 and overexpressing HER2 were assayed at the indicated timepoints following treatment with 1uM lapatinib.

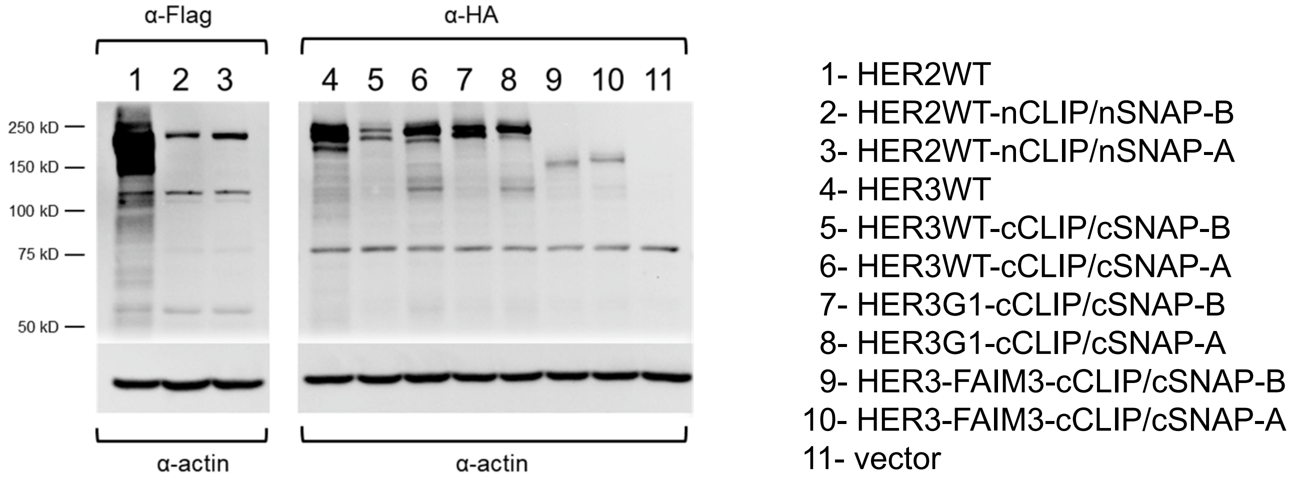

Figure S4. Expression of engineered constructs designed for complementation assays. Related to Figure 5. CHO cells were transiently transfected with the various constructs containing complementing halves of CLIP and SNAP tags to confirm their expression and sizes. The HER2 constructs (left panel) all have c-terminal Flag tags and they were immunoblotted using anti-Flag antibodies. The HER3 constructs (right panel) all have c-terminal HA tags and were immunoblotted using anti-HA antibodies. Lanes 1 and 4 are the non-engineered versions of HER2 and HER3.

index to microscopy images

Figure	construct design	fluorescent substrates SNAP (ICD)	CLIP (ECD)		
main article Figure 6B	Design A	green	red	controls	low magnification
main article Figure 6C	Design A	green	red	experimental arms	low magnification
Supplementary Figure 5A	Design B	green	red	controls	low magnification
Supplementary Figure 5B	Design B	green	red	experimental arms	low magnification
Supplementary Figure 5C	Design A	red	green	controls	low magnification
Supplementary Figure 5D	Design A	red	green	experimental arms	low magnification
Supplementary Figure 5E	Design A	red	green	experimental arms	high magnification
Supplementary Figure 5F	Design B	red	green	controls	low magnification
Supplementary Figure 5G	Design B	red	green	experimental arms	low magnification
Supplementary Figure 5H	Design B	red	green	experimental arms	high magnification

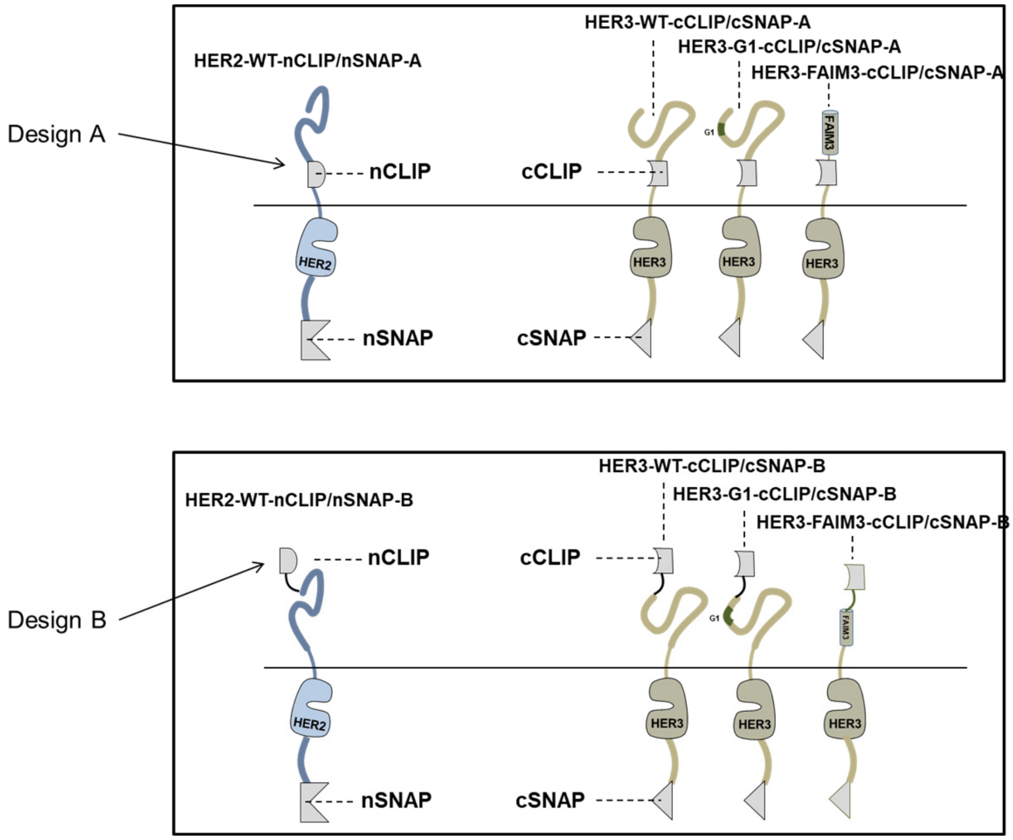

Figure S5. Microscopy images from various permutations, designs, and magnifications of the CLIP and SNAP complementation assays. Related to Figure 5.

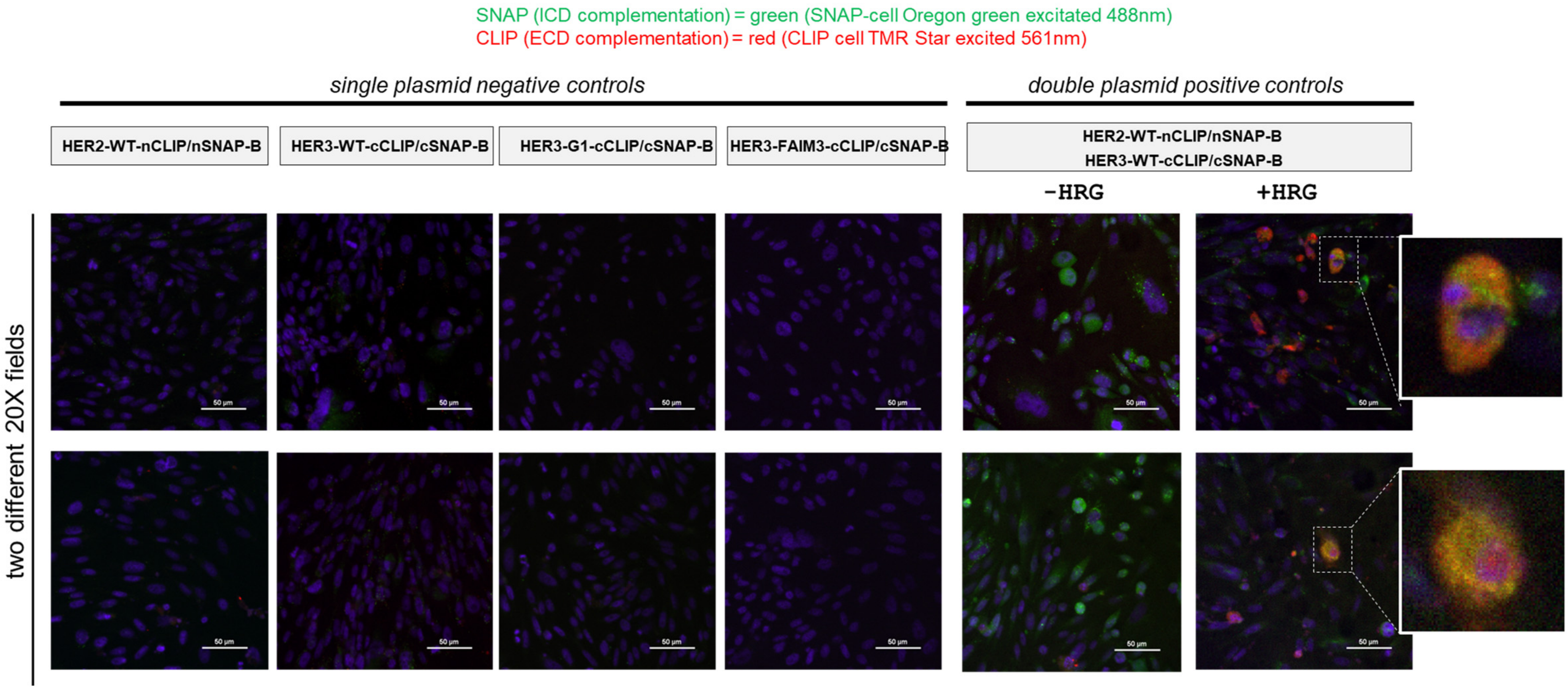

Figure S5A

CHO cells were transfected with the indicated constructs and complementation of the intracellular SNAP tags was visualized using SNAP-cell oregon green and complementation of the extracellular CLIP tags was visualized using CLIP-cell TMR (red fluorescence) and visualized by confocal microscopy with the appropriate laser excitations and filters. Blue fluorescence indicates hoechst staining of cell nuclei. The experiments shown here used constructs from design B. Related to Figure 5.

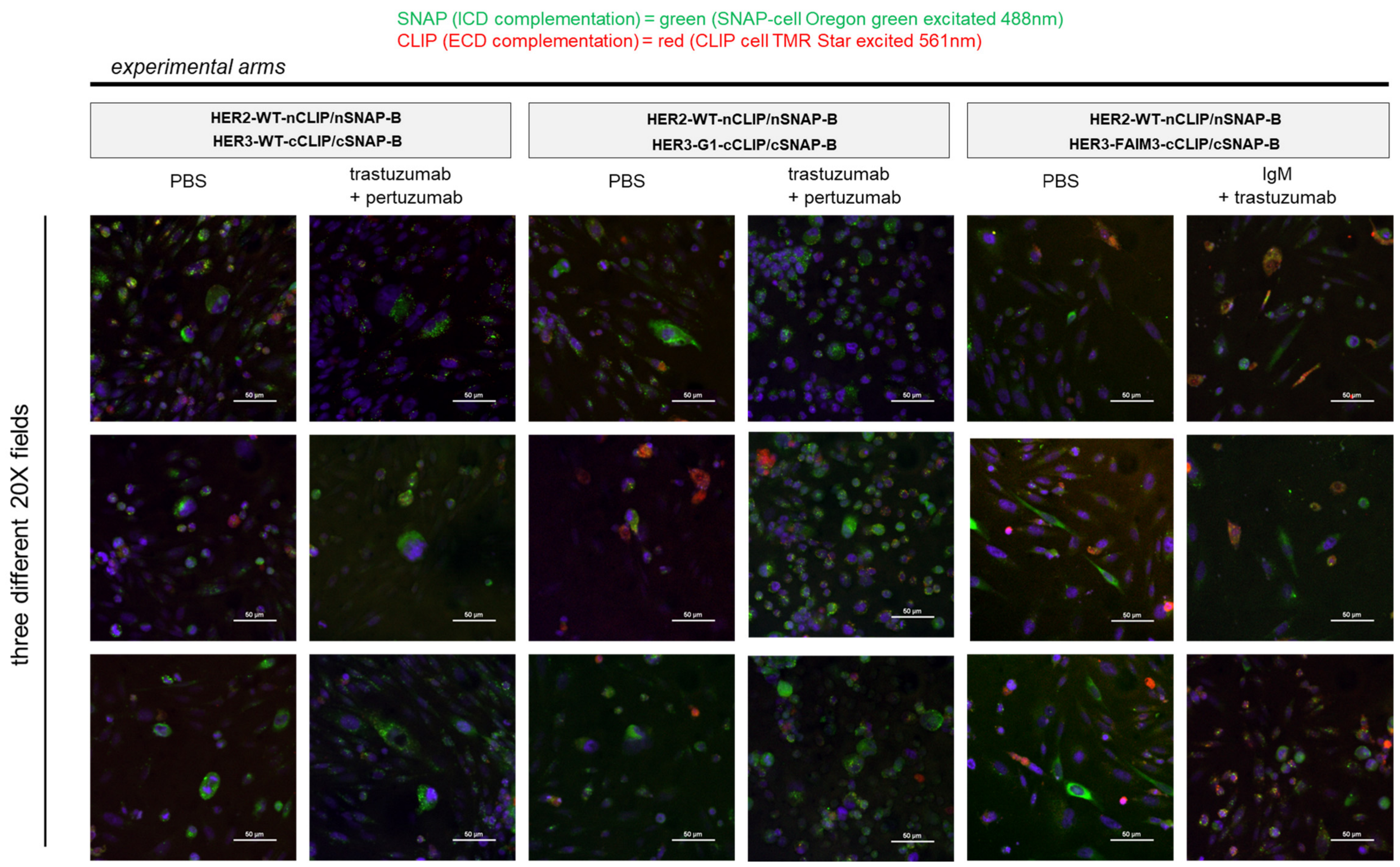

Figure S5B
CHO cells were transfected with the indicated constructs and complementation of the intracellular SNAP tags was visualized using SNAP-cell oregon green and complementation of the extracellular CLIP tags was visualized using CLIP-cell TMR (red fluorescence) and visualized by confocal microscopy with the appropriate laser excitations and filters. Blue fluorescence indicates hoechst staining of cell nuclei. The experiments shown here used constructs from design B. .Related to Figure 5.

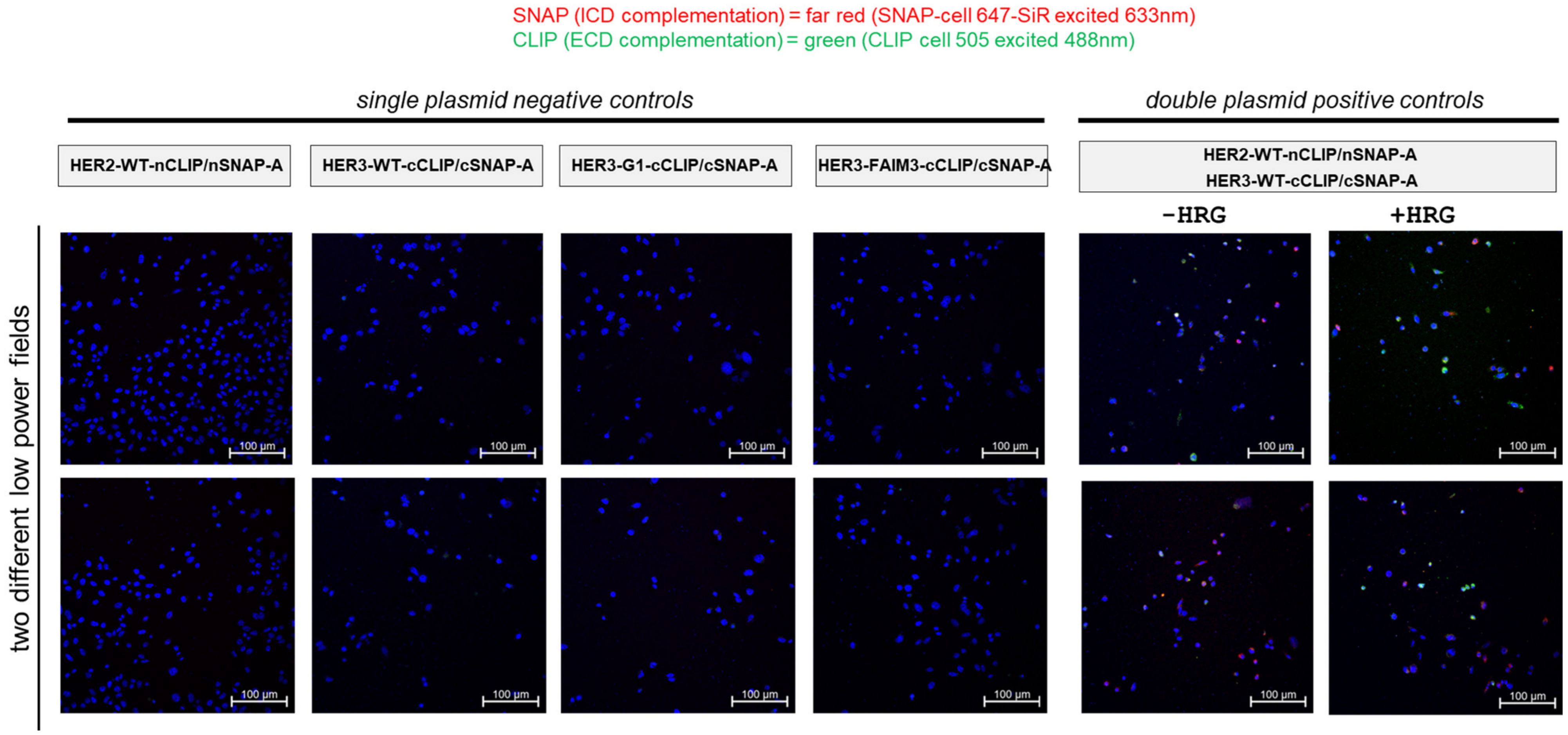

Figure S5C

CHO cells were transfected with the indicated constructs and complementation of the intracellular SNAP tags was visualized using SNAP-cell 647-SiR (far red) and complementation of the extracellular CLIP tags was visualized using CLIP-cell 505 (green) and visualized by confocal microscopy with the appropriate laser excitations and filters. Blue fluorescence indicates hoechst staining of cell nuclei. The experiments shown here used constructs from design A. Related to Figure 5.

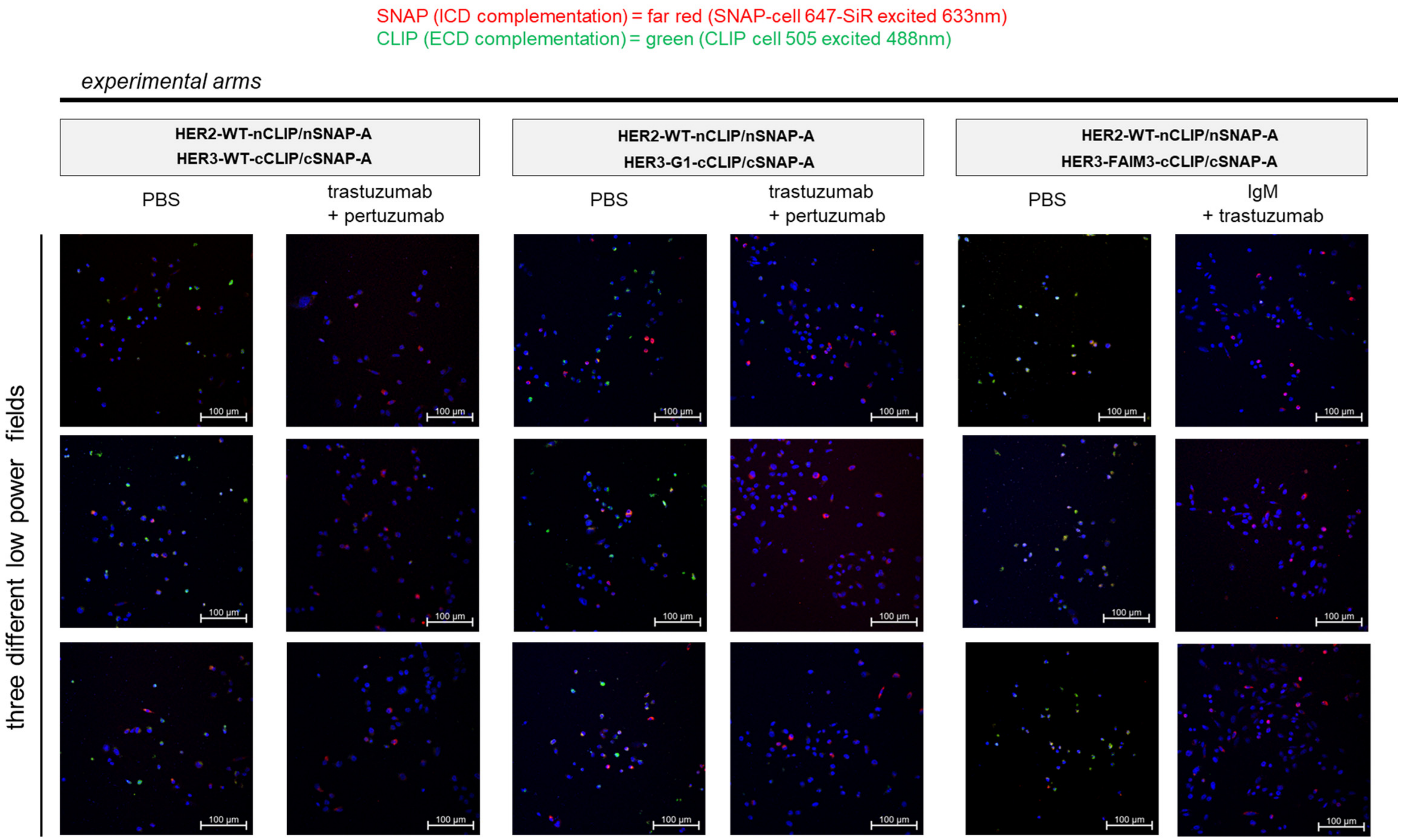

Figure S5D
CHO cells were transfected with the indicated constructs and complementation of the intracellular SNAP tags was visualized using SNAP-cell 647-SiR (far red) and complementation of the extracellular CLIP tags was visualized using CLIP-cell 505 (green) and visualized by confocal microscopy with the appropriate laser excitations and filters. Blue fluorescence indicates hoechst staining of cell nuclei. The experiments shown here used constructs from design A. Related to Figure 5.

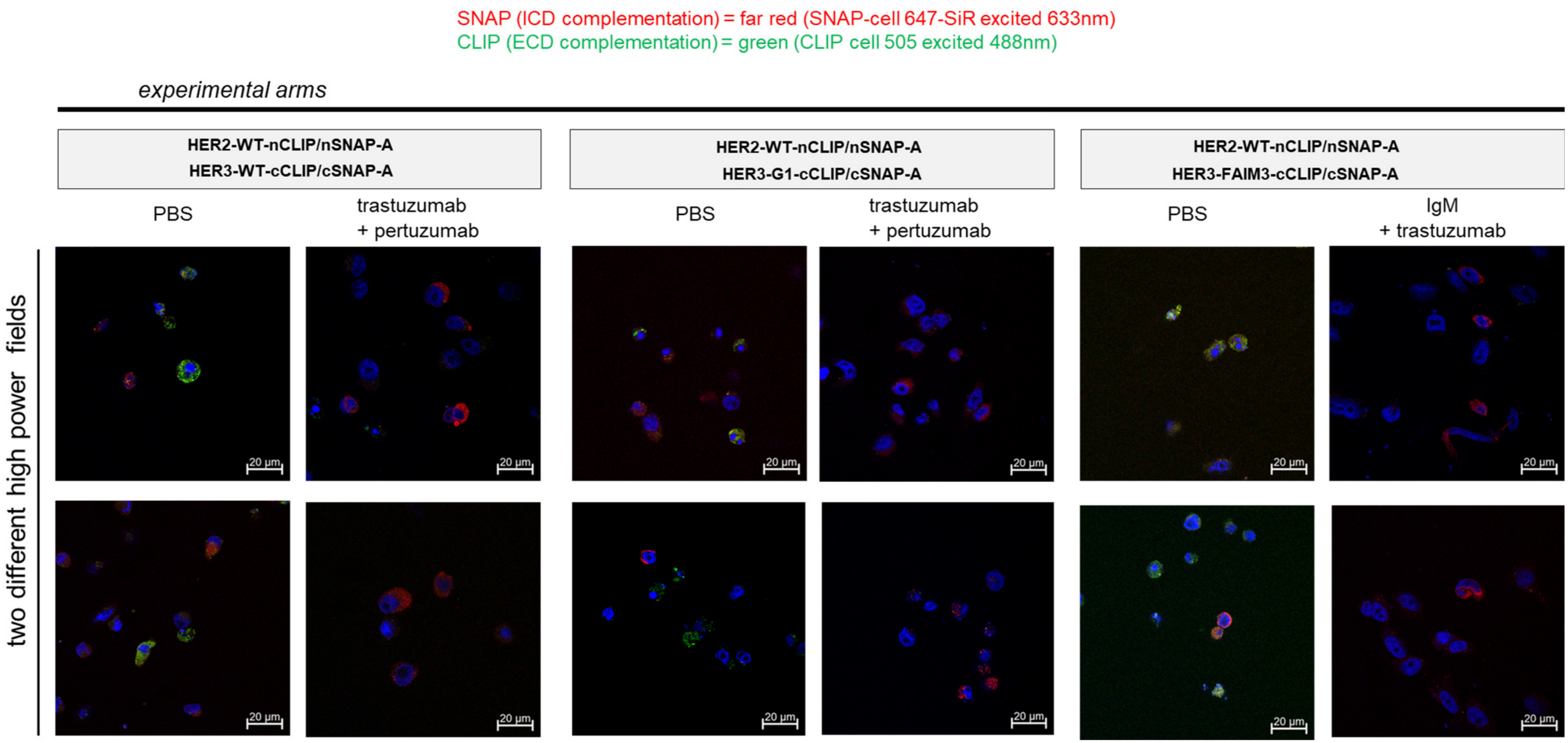

Figure S5E
CHO cells were transfected with the indicated constructs and complementation of the intracellular SNAP tags was visualized using SNAP-cell 647-SiR (far red) and complementation of the extracellular CLIP tags was visualized using CLIP-cell 505 (green) and visualized by confocal microscopy with the appropriate laser excitations and filters. Blue fluorescence indicates hoechst staining of cell nuclei. The experiments shown here used constructs from design A. Related to Figure 5.

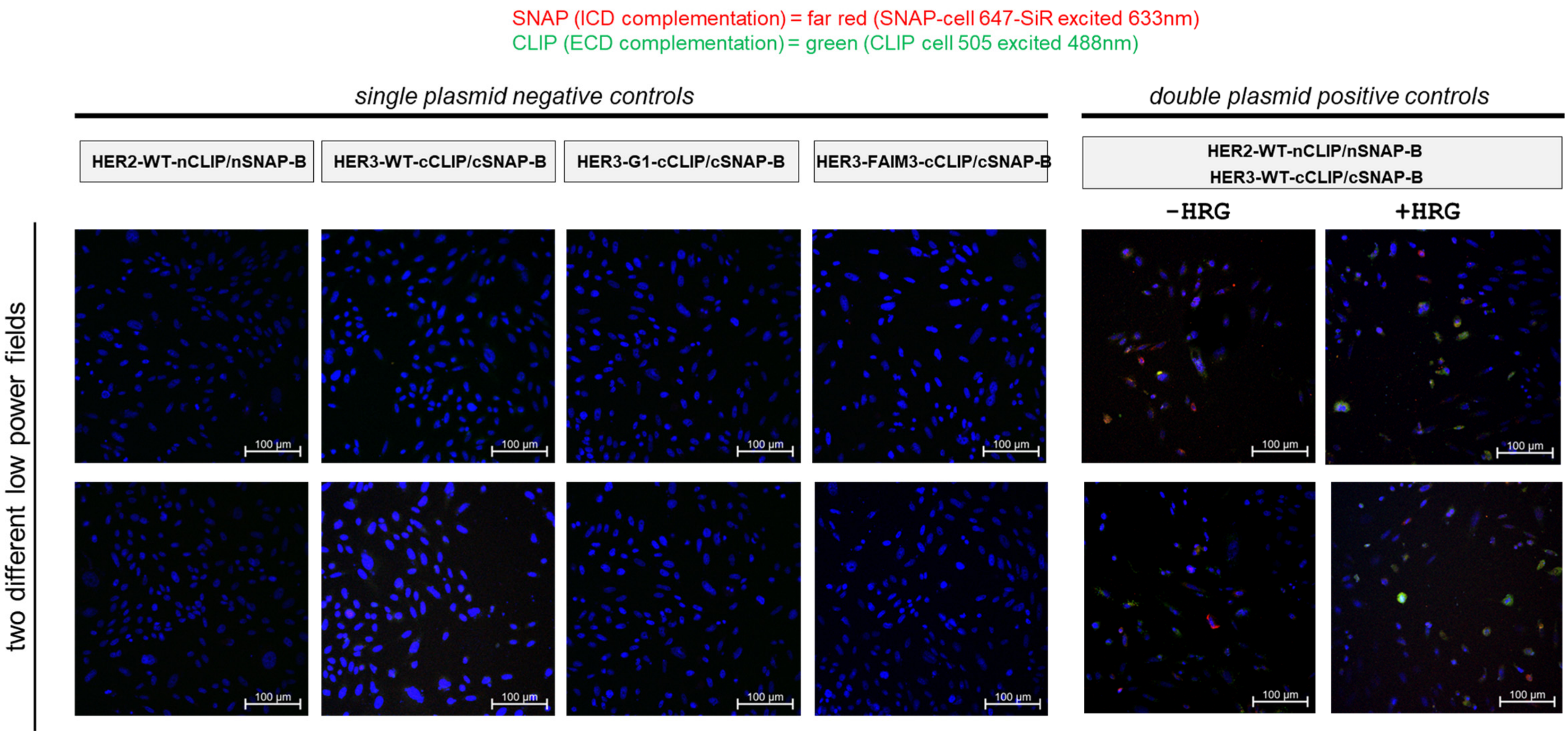

Figure S5F

CHO cells were transfected with the indicated constructs and complementation of the intracellular SNAP tags was visualized using SNAP-cell 647-SiR (far red) and complementation of the extracellular CLIP tags was visualized using CLIP-cell 505 (green) and visualized by confocal microscopy with the appropriate laser excitations and filters. Blue fluorescence indicates hoechst staining of cell nuclei. The experiments shown here used constructs from design B. Related to Figure 5.

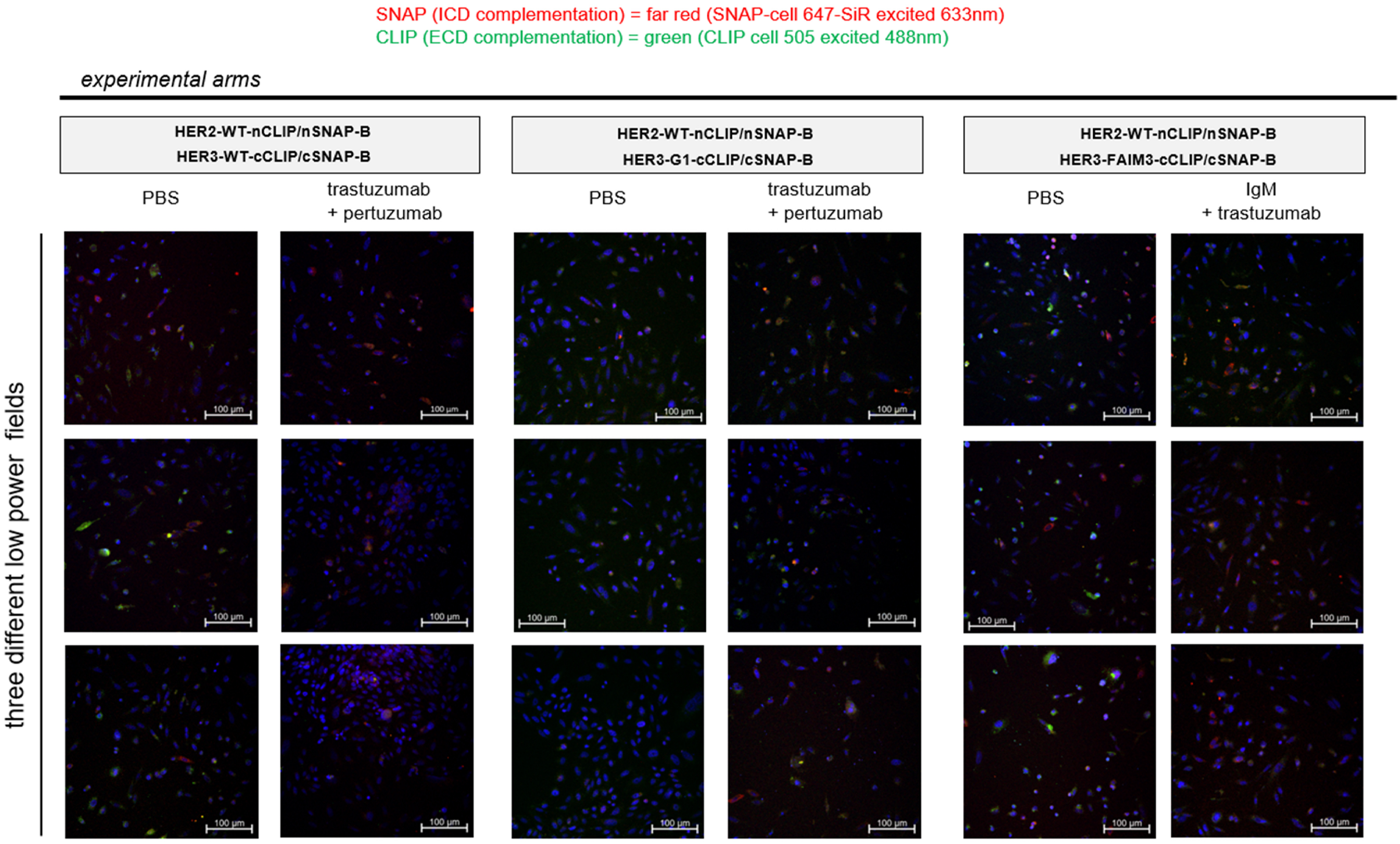

Figure S5G
CHO cells were transfected with the indicated constructs and complementation of the intracellular SNAP tags was visualized using SNAP-cell 647-SiR (far red) and complementation of the extracellular CLIP tags was visualized using CLIP-cell 505 (green) and visualized by confocal microscopy with the appropriate laser excitations and filters. Blue fluorescence indicates hoechst staining of cell nuclei. The experiments shown here used constructs from design B. Related to Figure 5.

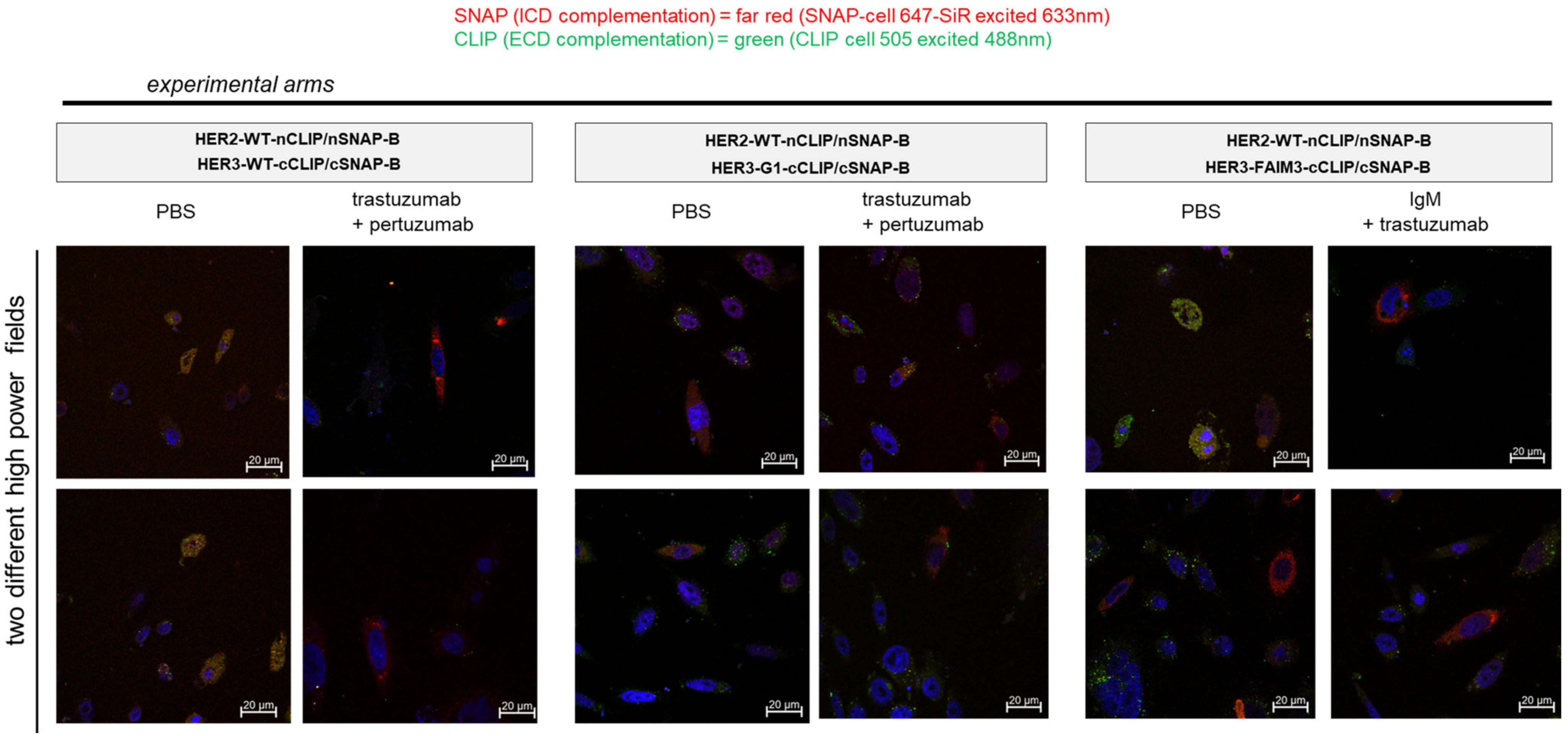

Figure S5H
CHO cells were transfected with the indicated constructs and complementation of the intracellular SNAP tags was visualized using SNAP-cell 647-SiR (far red) and complementation of the extracellular CLIP tags was visualized using CLIP-cell 505 (green) and visualized by confocal microscopy with the appropriate laser excitations and filters. Blue fluorescence indicates hoechst staining of cell nuclei. The experiments shown here used constructs from design B. Related to Figure 5.

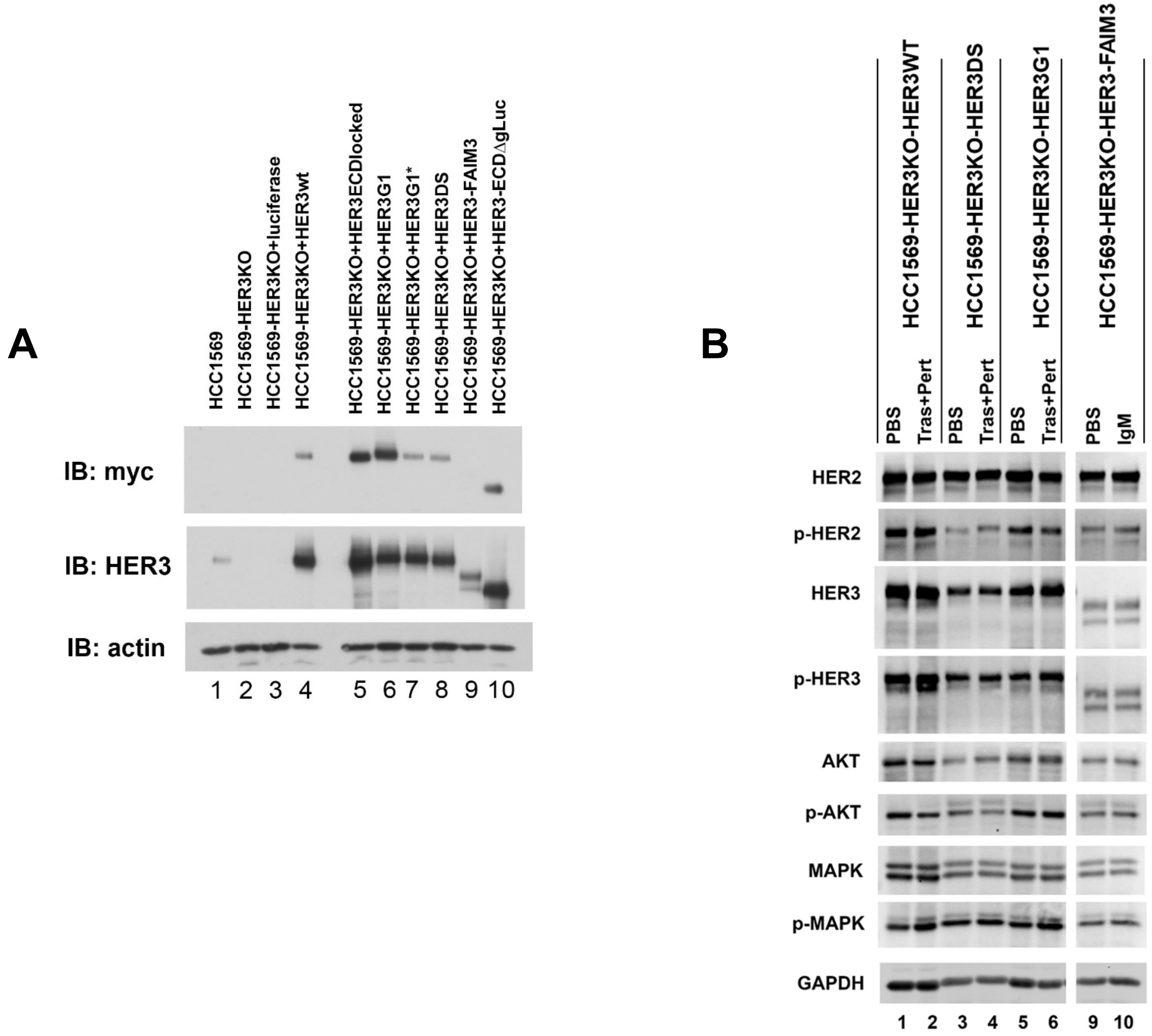

Figure S6. Expression of engineered HER3 constructs in HER2-amplified breast cancer cells. Related to Figure 7.

A) HCC1569 human HER2-amplified breast cancer cells were engineered to eliminate HER3 expression by Crispr/Cas targeting (HCC1569-HER3KO). To eliminate the role of clonal growth characterisitics in the replacement experiments, three separate clones of HCC1569-HER3KO cells were mixed together to generate a polyclonal HCC1569-HER3KO cell line (lane 2) and this cell line was used as the parental cell line for the various add-back experiments. These were then transduced to re-express wildtype HER3 (lane 4) or a variety of experimental mutant and hybrid HER3 constructs (lanes 5-10) described in this paper. The expression of firefly luciferase (lane 3) constitutes a negative control cell type. The add-back HER3 constructs all contain C-terminal myc tags except for the HER3-FAIM3 hybrid.

B) The indicated HCC1569-HER3KO add-back cells were treated with trastuzumab (50ug/ml) and pertuzumab (50ug/ml) or IgM (30ug/ml) for 4 hours or control PBS and the effects of drug treatment on signaling was assayed by immunoblotting as indicated. Lanes 7-8 of this experiment used mutants that are not pertinent to this paper and are not shown.

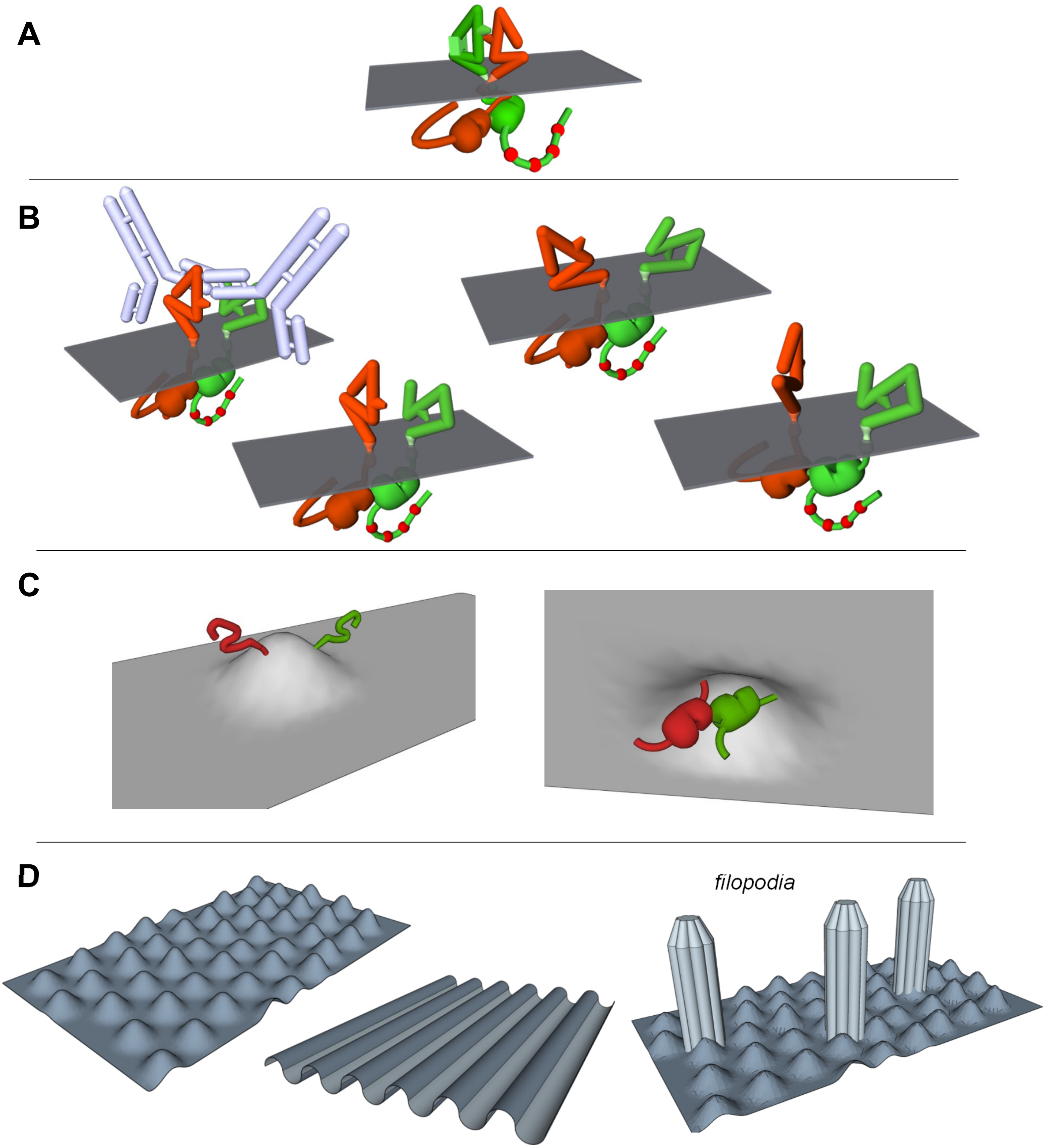

Figure S7. Schematic presentation of concepts in 3-dimensional layouts. Related to Figures 1-7.
A) Schematic showing the commonly envisioned mode of physiologic receptor dimerization on a flat membrane including the ligand-induced opening of the HER3 ECD, interaction of the ECD dimerization interfaces, and the asymmetric engagement of the kinase and juxtamembrane regions. HER3 is shown in green, HER2 in red. **B)** Potential conformational flexibilities within HER2 and HER3 that can account for a lack of ECD proximation and that can accommodate antibody binding to their ECDs. **C)** Kinase domain dimerization on a curved membrane driven by massive HER2 expression can occur free of all constraints imparted by their ECDs. Also see movie #2. **D)** Examples of various cell membrane surfaces that can provide the curvatures for ECD-ICD uncoupling.
